# Dietary Iron Intake and Biomarkers of Iron Status in Slovenian Population: Results of SI.Menu/Nutrihealth Study

**DOI:** 10.3390/nu14235144

**Published:** 2022-12-03

**Authors:** Živa Lavriša, Hristo Hristov, Maša Hribar, Barbara Koroušić Seljak, Matej Gregorič, Urška Blaznik, Katja Zaletel, Adrijana Oblak, Joško Osredkar, Anita Kušar, Katja Žmitek, Mitja Lainščak, Igor Pravst

**Affiliations:** 1 Nutrition Institute, Tržaška cesta 40, SI-1000 Ljubljana, Slovenia; 2 Biotechnical Faculty, University of Ljubljana, Jamnikarjeva Ulica 101, SI-1000 Ljubljana, Slovenia; 3 Computer Systems Department, Jožef Stefan Institute, Jamova cesta 39, SI-1000 Ljubljana, Slovenia; 4 National Institute of Public Health, Trubarjeva ulica 2, SI-1000 Ljubljana, Slovenia; 5 Faculty of Medicine, University of Ljubljana, Vrazov trg 2, SI-1000 Ljubljana, Slovenia; 6 University Medical Centre Ljubljana, Zaloška cesta 7, SI-1000 Ljubljana, Slovenia; 7 Faculty of Pharmacy, University of Ljubljana, Aškerčeva cesta 7, SI-1000 Ljubljana, Slovenia; 8 VIST–Faculty of Applied Sciences, Gerbičeva cesta 51A, SI-1000 Ljubljana, Slovenia; 9 Department of Internal Medicine, General Hospital Murska Sobota, Ulica dr. Vrbnjaka 6, SI-9000 Murska Sobota, Slovenia

**Keywords:** iron, intake, ferritin, haemoglobin, deficiency, Slovenia, EU Menu, Nutrihealth

## Abstract

Inadequate iron intake and iron deficiency are recognised as a public health problem in the population at large, and particularly in specific subpopulations. Dietary iron intake was analysed using data of the national Slovenian food consumption study, SI.Menu (*n* = 1248 subjects; 10–74 years), while iron status was evaluated with laboratory analyses of blood haemoglobin, serum ferritin, and iron concentration in samples, collected in the Nutrihealth study (*n* = 280, adults). The estimated daily usual population-weighted mean iron intakes ranged from 16.0 mg in adults and the elderly to 16.7 in adolescents, and were lower in females for all three age groups. The main dietary iron sources in all the age groups were bread and bakery products, meat (products), fruit, and vegetables. The highest prevalence of haemoglobin anaemia was observed in females aged 51–64 years (6.7%). Critically depleted iron stores (ferritin concentration < 15 µg/L) were particularly found in premenopausal females (10.1%). Factors influencing low haemoglobin, ferritin, and iron intake were also investigated. We observed significant correlations between iron status with meat and fish intake, and with iron intake from meat and fish, but not with total iron intake. We can conclude that particularly premenopausal females are the most fragile population in terms of inadequate iron intake and iron deficiency, which should be considered in future research and public health strategies.

## 1. Introduction

Iron deficiency (ID) is a common nutritional deficiency that affects around 2 billion people around the world, with iron deficiency anaemia (IDA) being among the most common consequences [1,2,3]. Iron deficiency, however, usually precedes IDA and is associated with poor exercise tolerance, headache, weakness, fatigue, dizziness, and shortness of breath, particularly in patients with chronic disease, but also in healthy people [4,5,6,7,8]. 

However, in the literature, ID is not always distinctive from IDA, and the two terms are often used interchangeably [9]. Besides ID, anaemia can also result from certain diseases and infections, as well as folate and vitamin B12 deficiency. Anaemia especially affects young children and pregnant women [1,3]. In the general population, premenopausal females are particularly at risk for ID due to menstrual losses; therefore, a somewhat higher daily iron intake is advised for this population group [10]. In athletes and those regularly involved in vigorous physical activity, ID is more common as well [11]. One of the causes for anaemia are also diseases that impair intestinal absorption, such as celiac disease. In the elderly, the common causes of ID are digestive diseases and digestive bleeding, which are often overlooked [12,13,14]. On the other hand, in Western countries, ID was not highlighted as a particular concern in adult males, where dietary iron intake is usually sufficient [15]. Excessive iron intake and body stores can be associated with a variety of chronic diseases, including cardiovascular disease [16], cancer [17], and diabetes [18,19]. 

The risk of insufficient dietary iron intake is often higher in developing countries, and in populations with mainly plant-based diets, due to the low iron bioavailability from such diets [20]. This effect can be further enhanced by drinking tannin-containing teas with meals [21]. In Europe, the major contributors of dietary iron are grains and meat/meat products [22]. The bioavailability of iron from various food sources can vary notably. While haem iron absorption can be up to 35%, non-haem iron is generally absorbed less effectively [23]. However, as there are no physiologically regulated means of iron excretion, iron absorption from foods is highly regulated, depending on the individuals’ iron status. This is supposed to have an even greater effect on the iron absorption than diet composition itself; therefore, individuals with lower iron stores tend to absorb more iron from foods, even from non-haem iron sources [24,25]. Besides the iron status, other health characteristics of the individuals, particularly obesity, play a key role in the amount of the iron absorbed from the diet [26]. Alcohol consumption was also shown to affect iron stores in the body [27]. The total bioavailability of iron from a meal depends on many factors, and the enhancing and inhibiting activities of single compounds are not the only factors influencing iron absorption [26]. Daily iron turnover is 1–3 mg, and iron body stores account for 2–3 g; therefore, the dietary compensation for iron loss can take time [28].

Slovenia adapted the D-A-CH (Germany, Austria, and Switzerland) dietary recommendations [29], where the recommended iron intake is set as follows: adolescent males: 12 µg/day; adolescent and premenopausal females: 15 µg/day; adult and elderly males, and postmenopausal females: 10 µg/day [30]. The European Food Safety Authority (EFSA) established population reference intakes (PRI) at 11 µg/day for males, postmenopausal and adolescent females (10–11 years); 13 µg/day for adolescent females (12–17 years), and 16 µg/day for premenopausal females (18–50 years). The average requirements (AR) are set in a way such that half of the population meets the set threshold, which are, according to EFSA, 8 µg/day for adolescent males (10–17 years) and females (10–11 years), 7 µg/day for adolescent and premenopausal females, and 6 µg/day for adult males and postmenopausal females [22]. 

Until now, iron intake and status in Slovenia were only studied in specific population subgroups [5,31,32,33,34,35,36], and no nationally representative data are available. Literature data from studies in European countries show that dietary iron intakes were generally higher in males than females for all age groups [22]. Around 80% of European males were reported to meet recommendations for dietary iron intakes, even up to 22.7 mg/day, which is linked to a high meat consumption [15]. On the other hand, much lower dietary iron intakes are reported in females. In many European countries, women of reproductive age had dietary iron intakes of below 15 mg/day, contributing to the low iron status in this population group, which is particularly at risk for ID [37]. 

However, to assess the body iron status, low dietary iron intake cannot be used as the criteria for an assessment of deficiency, because it is affected by other different factors [38]. Common markers in the assessment of iron status are serum iron, transferrin, transferrin saturation, and ferritin [39]. Ferritin is considered to be the most sensitive and specific marker for ID assessment [1], as in healthy individuals, it indicates iron stores in the body, which can be used for the production of erythrocytes. Ferritin levels of below 15 μg/L are considered as critical by the World Health Organisation (WHO), and indicate depleted bone marrow iron stores [3]. In clinical practice, ID is often ascertained at ferritin levels below 30 µg/L [14,40]. As serum ferritin is known as an acute-phase protein due to its significant increase in the presence of inflammation and certain chronic disease, such as cancer [41], a cut-off ferritin level for ID is sometimes increased up to 100 μg/L [42]. In the presence of chronic inflammation conditions, with ferritin levels of 100–300 μg/L, transferrin saturation (TSAT) can be also considered, with levels below 20% being diagnostic of ID [43]. Serum iron levels ≥13 μmol/L are generally not concerning in terms of ID, but this parameter can vary significantly, depending on the past days’ diet and circadian rhythms [44]. In clinical practice, haemoglobin is typically used as a diagnostic parameter for anaemia. Haemoglobin in red blood cells presents a major iron pool in the body, where it serves as an oxygen transporter [45,46,47]. According to the WHO, haemoglobin concentrations <120 g/L in adolescents and adult females and <130 g/L in adult males indicate the presence of anaemia [48]. 

In Europe, especially children, premenopausal and pregnant women are reported to be at risk for ID, while males are generally not considered as a vulnerable population [49]. The prevalence of ID in premenopausal females accounts for 10–30% of the population, with IDA rates ranging over 1.5–14%, depending on the cut-off criteria used [49,50]. Mean serum ferritin levels typically range over 26–38 μg/L and vary among countries [50].

In Europe, iron fortification of foods is currently not a general practice, as seen in some other countries, and is mainly voluntary, with regulated forms of iron sources [51]. In Slovenia, the food category with the highest proportion of iron enriched products is breakfast cereals (39%) [52].

The objective of the present study was to estimate the daily dietary iron intake in Slovenian adolescents, adults, and the elderly population with foods, and to analyse selected biomarkers of iron status in the adult and elderly populations. Data were obtained from a nationally representative food consumption study (SI.Menu), supplemented with analyses of biological biomarkers on a sub-sample of adults and elderly (Nutrihealth study). We also investigated the main dietary sources of iron, and explored the determinants linked to low iron intake and status.

## 2. Materials and Methods

### 2.1. Subjects and Study Design

A cross-sectional SI.Menu study was performed on a nationally representative sample of the Slovenian population (a total population of approximately 2 million) between March 2017 and April 2018. Study design, data collection, and sampling were performed in accordance with the European Food Safety Authority (EFSA) Guidance on EU Menu Methodology [53]. Details of the SI.Menu study have been previously published elsewhere [54]. The selection of participants was random and was obtained from the Central Register of Population of Slovenia, considering their age, sex, and residential area. Institutionalised people were not included in the study. The study protocol was approved by the National Medical Ethics Committee (KME 53/07/16; approval No. 0120-337/2016 issued on 19 July 2016). Prior to inclusion in the study, all subjects were informed about the study and signed an informed consent. For adolescents, informed consent was obtained from their parent or legal guardian. The SI.Menu study sample included 2280 participants, which were further divided according to their age: adolescents (10–17 years), adults (18–64 years), and elderly (65–74 years). The response rate was 62% [54]. After exclusion of subjects with missing information and under/over-reporters (see Section 2.5), the final study sample included N = 1248 participants. 

**Figure 1:**
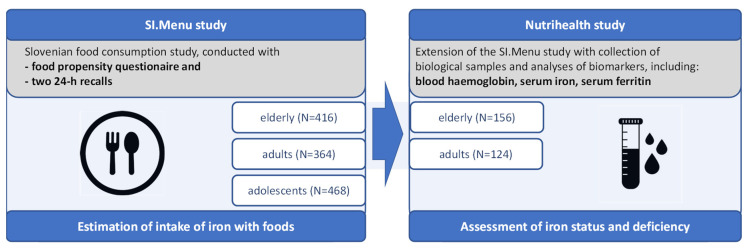
Schematic presentation of SI.Menu and Nutrihealth studies.

Additionally, 280 participants (*n* = 125 adults and *n* = 155 elderly) from the SI.Menu study further participated in the Nutrihealth study, where blood and urine samples were collected (Figure 1). Details on the Nutrihealth study methodology were also published previously [55]. The protocol of this supplementary study was approved by the Slovenian National Medical Ethics Committee, Ljubljana, Slovenia (KME 72/07/16; approval No. 0120-337/2016-4 issued on 7 July 2017). For the purpose of the present study, haemoglobin, serum iron, and serum ferritin concentrations were determined in the blood samples. 

### 2.2. Data Collection

Data for the SI.Menu study were collected through two interviews. A general questionnaire, anthropometric data, food propensity questionnaire (FPQ) data, and first 24 h dietary recall were collected in the scope of the first interview, while in the second interview (which took place 7–21 days after the first one) a second 24 h dietary recall was performed. Data for biomarkers were collected with laboratory analyses of the blood samples and collected with the Nutrihealth study.

#### 2.2.1. General Questionnaire

The general questionnaire is explained in detail elsewhere [54]; the questionnaire included sociodemographic and socioeconomic data, including place of residency, education level, employment, self-reported financial status, smoking, physical activity level, presence of chronic disease, and specific dietary patterns. Participants’ body height and body weight were measured for body mass index (BMI) calculation. 

#### 2.2.2. Dietary Records and Iron Intake

Two 24 h dietary recalls were performed 7–21 days apart, with a 71% inclusion of workdays and 29% of weekends. Dietary recalls were performed by a trained researcher. Respondents reported on the foods that they had consumed in the previous day, estimating the portions using a nationally validated picture book of commonly consumed foods, along with their portion sizes [56]. The usual consumption frequency of specific food categories in the past 12 months was estimated through FPQ [54]. 

Data collected in the dietary recalls were analysed using the Open Platform for Clinical Nutrition (OPEN) [57]—a web application based on the Slovenian food composition database, enabling the calculation of dietary intakes of macro- and micronutrients. For foods of which data on iron content were not available in the OPEN, this was searched for in other databases, particularly in the Finnish National Food Composition Database [58] and the United States Department of Agriculture Food Composition Database (USDA) [59]. As in our previous study [60], the estimation of the usual daily intakes was conducted with the consideration of FPQ using the Multiple Source Method (MSM) [61]. We calculated total usual daily iron intake (not including medicines and supplements), and daily iron intake from meat and fish products.

#### 2.2.3. Iron Status

Fasting blood samples were provided by the participants of the Nutrihealth study, and haemoglobin, serum iron, and serum ferritin concentrations were determined at University Medical Centre, Ljubljana, Slovenia. 

Haemoglobin was analysed using the ADVIA 2120/2120i haematology system, which uses a cyanide-free haemoglobin method. This is a two-step procedure. In the first step, red blood cells are lysed to release haemoglobin, In the second, the haem iron in the haemoglobin is oxidised from the ferrous to the ferric state, coordinating one hydroxide ion and one water molecule as an axial ligand to form monoaquomonohydroxyferri-porphyrin as the reaction product. The optical readings were obtained calorimetrically at 565 nm. Performance characteristics: linearity 0–225 g/L, precision <1% (CV = 0.93%). 

Serum ferritin concentration was measured with the chemiluminescence immunoassay determined on an Immulite 2000 XPi analyser (Siemens Healthineers, Gwynedd, UK). Performance characteristics for the assay are as follows. The ferritin limit of detection is 0.4 µg/L, and the linearity of the assay is in the range from 0.4 to 1500 µg/L, with a recovery range of 90 to 106%, standardised in terms of the WHO 2nd IS 80/578. The intra-assay and inter-assay coefficients of variation ranged from 3.0% to 5.3% and from 4.0% to 7.2%, respectively. 

Serum iron was determined on the ADVIA 2400, which uses a method based on a ferrozine method. The iron is released from transferrin under acidic conditions and is reduced to its ferrous state to combine with a chromogen for colorimetric measurement. The coloured chromophore absorbs at 571/658 nm. Performance characteristics: the analytical range for serum is 0.3–179 µmol/L, and the precision is 1.5–2.2% within-run and 1.1–3.0% between-run.

### 2.3. Covariates

Depending on the type of analysis and the outcome variable, a variety of predictors of iron intake and iron status were used: sex (male, female), residential area (self-declared: rural, intermediate, urban), education level (adults, elderly: university degree, no university degree), financial status (adults and elderly: below/above monthly average income of 1300€), and BMI (normal, overweight); physical activity using the IPAQ (International Physical Activity Questionnaire) score (low intensity, moderate, high intensity); smoking status (non-smoker, current/occasional/ex-smoker), employment status (adults: employed, unemployed, retired, student); chronic disease (present, not present), recent disease diagnosis (present, not present), supplement use (user, non-user), tea consumption (<3× week/never, other), alcoholic beverage consumption (user, non-user), diet (no special diet, medical/weight loss diet), behavioural diet (no diet, vegetarian/vegan), serum ferritin status (above/below 30 µg/L), and serum iron status (above/below 13 µmol/L). 

In adult females, participants were further categorised based on their age, to gain an insight into the pre- and post-menopausal subgroups. Participants with BMI > 25 kg/m^2^ were considered overweight, and all of the participants below this cut-off point were appointed to a group with normal body weight (because only a few participants were underweight). For adolescents, overweight status was determined using sex-/age-adjusted cut-off points (>1 standard deviation (SD)) [62,63]. The IPAQ score was calculated based on data of self-reported physical activity [64]. A recent disease was considered as a disease diagnosed within the past 12 months. The included diseases were high blood pressure (>140/90 mm Hg), high cholesterol (>5 mmol/L), high blood sugar (>6.1 mmol/L), diabetes type 1, diabetes type 2, myocardial infarction, chest pain, heart failure, brain infarction, peptic or duodenal ulcer, liver cirrhosis, kidney disease, cancer of large intestine, thyroid disease, and osteoporosis. 

### 2.4. Definition of Cut-Offs for Iron Intake and Status

Analyses of the iron intake were determined using the following cut-off values: D-A-CH recommendations [29], implemented into the Slovenian national reference intakes (DRI): adolescent males: 12 µg/day, adolescent and premenopausal females: 15 µg/day; adult and elderly males, postmenopausal females: 10 µg/day [30]. The EFSA’s population reference intakes (PRI): 11 µg/day for males, postmenopausal and adolescent females (10–11 years); 13 µg/day for adolescent females (12–17 years), and 16 µg/day for premenopausal females (18–50 years). The EFSA’s average requirements (AR): 8 µg/day for adolescent males (10–17 years) and females (10–11 years), 7 µg/day for adolescent and premenopausal females, and 6 µg/day for adult males and postmenopausal females [22].

For expressing the prevalence of a low concentration of each of the biomarkers, the following cut-off values were used: haemoglobin: <120 g/L and <130 g/L [48]; serum ferritin: <15 µg/L [65], <30 µg/L, <100 µg/L [42], >300 µg/L, and >500 µg/L [66]; serum iron: <13 µmol/L [44].

### 2.5. Data Analysis

For each of the two dietary recalls, data cleaning was performed separately. The assessments of under- and over-reporting were previously described [67]; we excluded subjects with reported energy intakes <500 kcal/day, and analysed under- and over-reporting with the Goldberg method [68], based on the ratio of reported daily energy intake and basic metabolic rate (BMR). The usual dietary iron intakes were calculated from two 24 h dietary recalls and FPQ. The data were modelled using the Multiple Source Method (MSM), with age, sex, and BMI being considered as covariates. This enabled the provision of data on the usual dietary intake by correcting the data variation of iron intake in individuals via FPQ [69]. 

To assure nationally representative results, census data from the year 2017 were used for population weighting for each of the three cohorts (by age and sex), using the iterative proportional fitting method [70]. For each age cohort, a population-weighted mean iron intake was calculated, and the prevalence of inadequate daily iron intake based on different recommendations was determined. For all population groups, we also calculated the mean population-weighted concentrations of haemoglobin, serum ferritin, and serum iron, and the prevalence according to different cut-offs.

Foods, reported in the 24 h recalls, were categorised according to the modified categorisation system published by the Global Food Monitoring Initiative [71], and the relative contributions of specific food categories in total daily iron intake were presented for each food category. 

Participants were also divided into subgroups based on interquartile group analysis on the consumption of particular food categories (meat and fish products, bread and bakery products, vegetable and vegetable products, and cereal and cereal products). QR: 0–25%: lower consumption of the selected food category; and QR: 75–100%: higher consumption of the selected food category. Additionally, the mean total dietary iron intake and serum ferritin concentration for subgroups of participants of the Nutrihealth study was determined.

For the assessment of iron intake and haemoglobin concentration, multiple linear regression analysis was used. Predictor variables in the iron intake models were sex, residential area, education, financial status, BMI, IPAQ, employment status, smoking status, and diet type, while in the haemoglobin concentration model we also considered recent/past disease, the use of food supplements, dietary iron intake, the consumption of alcoholic beverages, serum ferritin, and iron status. The prevalence of inadequate dietary iron intake (according to D-A-CH cut-off values) and the prevalence of low ferritin concentration (<30 µg/L for adults and the elderly, and <100 µg/L for the elderly) were analysed via logistic regression analyses using the above-mentioned predictor variables. Unadjusted means and crude odds estimates were also determined.

Correlation analyses were conducted to provide further insights on dietary iron intake and iron status. Analyses were done using the Nutrihealth study sample, with the exclusion of subjects that reported using iron food supplements. Analyses were done on merged sample of adults and elderly, and on a more homogenous sample of women 18–50 years. Correlation plots are presented for associations of (a) total iron intake, (b) meat and fish intake, and (c) iron intake from meat and fish, with (1) haemoglobin concentration and (2) ferritin concentration.

STATA (version 17.0; StataCorp LLC, College Station, TX, USA) was used for statistical analyses. Statistical difference was reported at *p* < 0.05. Marginally significant differences are mentioned at *p* < 0.1.

## 3. Results

The characteristics of the SI.Menu study sample (N = 1248) of adolescents, adults, and the elderly are presented in Supplementary Table S1.1. About a quarter of the participants reported the use of multivitamin supplements; however, specific data on the iron supplementation were not available. Altogether, 34% adults and 37% of the elderly from the SI.Menu study were further included in the Nutrihealth study (N = 280), where blood samples were also collected. The demographic characteristics of the Nutrihealth study sample are presented in Supplementary Table S1.2.

The distribution of the usual total daily iron intake is presented in Supplementary Figure S1, and population-weighted descriptive statistics for iron intake are presented in Table 1. In adolescent females, and in females below the age of 50, the mean iron intake was just under the recommended 15 mg/day (14.7 mg/day vs. 14.1 mg/day, respectively), while in males of all age groups, the mean daily iron intake was above the reference daily iron intake (12 mg/day for male adolescents and 10 mg/day for male adults and elderly): for adult and elderly males, the population-weighted mean iron intake was 17.7 mg/day in adult and 17.1 mg/day in elderly males, while in adolescent males, it was 18.4 mg/day. The prevalence of inadequate daily iron intake was found in more than 70% of adolescent females and females below 50 years of age, while in elderly females it was 20.7%. However, for adult and elderly males, inadequate daily iron intake was observed in less than 10% of the population. Interestingly, mean iron intakes calculated per 1000 kcal/day were very similar in all age groups and sexes (approximately 6 mg) (Table 1). 

Using logistic regression analyses, we investigated predictors associated with inadequate daily dietary iron intake (Table 2). The cut-offs used were based on the national reference values for specific age group/sex [30]. In all age groups, sex was found as a significant predictor of inadequate daily dietary iron intake (*p* < 0.001). The highest odds for inadequate daily dietary iron intake were seen in adult females below 50 years (OR 51.2; CI: 20.6–127.6). In adolescents, smoking status was also found to be a significant predictor of iron intake, with smokers having higher odds for inadequate iron intake than non-smokers (OR 3.66; CI: 1.51–8.88; *p* < 0.01). In adults, we observed a lower risk for inadequate iron intake in those having a BMI above the normal range (OR 0.48; CI: 0.24–0.97; *p* < 0.05). A significantly higher risk for inadequate iron intake was found in the elderly, with a moderate activity score (IPAQ) (OR 1.99; 0.98–4.03; *p* = 0.05). For vegetarians and vegans, a trend of higher risk for inadequate iron intake was noticed in all age groups, although it could not be statistically confirmed through a modelling approach due to the low sample size.

**Figure 2:**
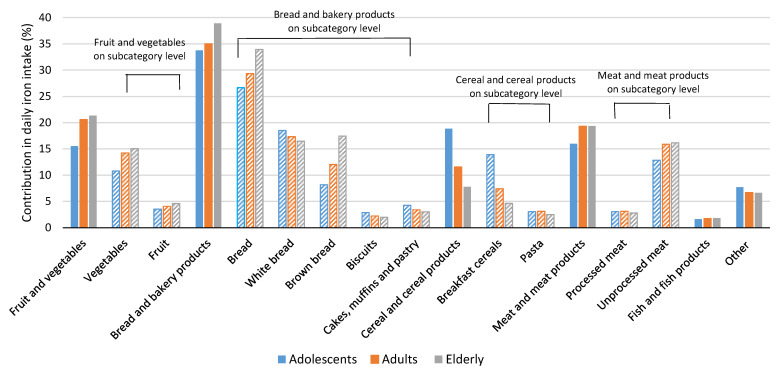
Relative contribution of selected food categories (bars with solid pattern) and subcategories (bars with lined pattern) to usual daily dietary iron intake among adolescents, adults, and elderly (% of total iron intake).

Figure 2 and Supplementary Table S3 show the relative contributions of different food categories to daily iron intake. The food category with the most important contribution of iron in the daily diet was bread and bakery products, with contributions of above 30% in all age groups, followed by meat and meat products (especially unprocessed meat), and fruits and vegetables. In adults and the elderly, the category of fruits and vegetables had a more notable contribution to iron intake (20.6% and 21.3%, respectively) than in adolescents, for whom cereal and cereal products were more important iron contributors (18.8%) than fruits and vegetables (15.5%).

A sub-sample of adult and elderly participants from the SI.Menu study further participated in the Nutrihealth study, where biological samples were taken to investigate diet related biomarkers. In previous papers, we focused on biomarkers related to vitamins (vitamin D [55], folate [60], and vitamin B12 [72]), while in the present study, we investigated markers of iron status, using serum ferritin, haemoglobin, and serum iron concentrations. The study results are presented in Table 3. The population-weighted mean haemoglobin concentration was above the WHO threshold for low haemoglobin (120 g/L for females and 130 g/L for males) in both studied populations—154.0 g/L vs. 152.0 g/L in adult and elderly males, respectively, and 139.0 g/L vs. 138.9 g/L in adults and elderly females, respectively. Based on the above-mentioned thresholds, a notable prevalence of low haemoglobin was observed only in females aged 51–64 years (6.9%) and elderly males (4.1%). The mean population-weighted serum ferritin concentration was 222.6 µg/L in elderly males, while in females (18–50 years) it was 68.2 µg/L. It should be highlighted that 27.2% of females from this age group had serum ferritin concentrations <30 µg/L, and 10.1% also had concentrations below 15 µg/L. A regression analysis of factors associated with serum ferritin status below 30 µg/L (Supplementary Table S4) showed inadequate iron intake (OR 0.2; CI: 0.3–1.0; *p* < 0.05) as a significant predictor in adults, and education (OR 3.8; CI: 0.8–17.1; *p* < 0.1) as being marginally significant in the elderly. For the elderly, regression analysis was also performed using threshold ferritin concentration <100 µg/L (Supplementary Table S5), where only sex was found as a significant predictor—indicating higher odds in females. We also investigated a population-weighted prevalence of serum ferritin concentration above 300/500 µg/L (Table 3). Altogether, 17.7% of adult males and 22.4% of elderly males, but very few females, had serum ferritin concentrations >300 µg/L. 

To investigate the associations of sociodemographic factors with haemoglobin concentrations in adults and the elderly, we used multiple linear regression modelling on a sample of participants of the Nutrihealth study (Supplementary Table S6). Significant factors associated with haemoglobin concentration in adults were sex (*p* < 0.001), education (*p* < 0.05), the consumption of alcoholic beverages (*p* = 0.05), serum ferritin status (*p* < 0.001), and serum iron status (*p* < 0.05). In elderly, sex (*p* < 0.001), medical diet (*p* < 0.001), serum ferritin status (*p* < 0.001), and serum iron status (*p* < 0.05) were found to be significant, while residential area and the consumption of alcoholic beverages were found to be marginally significant at *p* < 0.1. The analysis of the mean haemoglobin concentration within each of the predictor levels showed significantly higher haemoglobin levels in adult and elderly males. Adults and the elderly who had serum ferritin concentration above 30 µg/L had significantly higher mean haemoglobin concentrations. This was also observed in adults and the elderly with serum iron concentrations >13 µmol/L.

**Figure 3:**
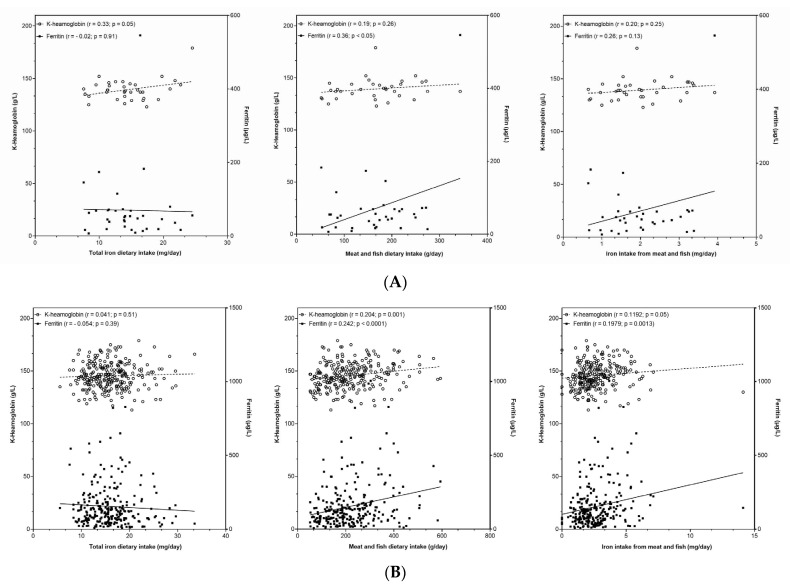
(**A**) Association between haemoglobin and ferritin concentration with (**left**) total iron dietary intake, (**centre**) meat and fish dietary intake, and (**right**) iron intake from meat and fish in Nutrihealth sample, with exclusion of subjects using iron food supplement. (**B**) Association between haemoglobin and ferritin concentration with (**left**) total iron dietary intake, (**centre**) meat and fish dietary intake, and (**right**) iron intake from meat and fish in Nutrihealth sample, with the exclusion of subjects using iron food supplement.

With the consideration of challenges in the iron status in adult premenopausal females aged 18–50 years, we further focused on this group and investigated the associations of iron status biomarkers with usual total iron intake. Analyses were done on a Nutrihealth study sample after excluding subjects which reported the use of iron containing food supplements (Supplementary Table S7). Figure 3A shows a plot with the association of usual iron intake with haemoglobin and serum ferritin. A positive correlation between iron intake and haemoglobin was found (r = 0.33, *p* = 0.05), while no correlation was found with ferritin (*p* = 0.91). To provide further insights, we also checked associations for iron intake with meat and fish products. The association with ferritin was positive while slightly missing the marginal significance level(r = 0.28, *p* = 0.13), with consideration of the relatively small sample size. Another correlation analysis was therefore performed with meat and fish intake (instead of iron intake), where statistically significant correlation with ferritin was observed (r = 0.38, *p* = 0.05) (Figure 3A). The same correlation analysis was conducted for the merged Nutrihealth sample of adults and elderly (for males and females, with exclusion of users of iron containing food supplements) (Figure 3B). Despite a very heterogenous sample, we observed strongly significant positive correlations between ferritin and both meat and fish intake, and iron intake from meat and fish (*p* = 0.001 and 0.0001, respectively), but not with total iron intake (*p* = 0.39). Similar but somewhat less significant associations were found with haemoglobin, but again only for meat and fish intake, and iron intake from meat and fish.

**Figure 4:**
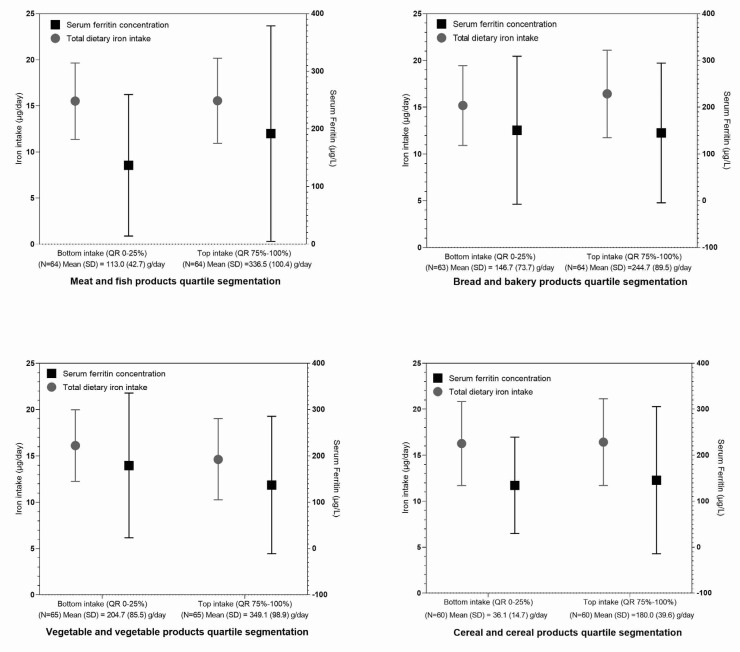
Mean total dietary iron intake and serum ferritin concentration for participants of the Nutrihealth study not supplementing iron, based on their usual daily consumption of foods from the selected food categories. Interquartile groups are indicated as follows: lowest intake (QR: 0–25%)—lower consumption of foods in the selected food category; highest intake (QR: 75–100%)—higher consumption of foods in the selected food category.

These observations lead us to further analyses of the dietary patterns in relation to major iron sources. While study subjects with ferritin levels below and above 100 µg/L had similar usual dietary iron intakes, the group with higher ferritin levels had a higher proportion of dietary iron intake from meat and fish products (Supplementary Table S8), which is in line with results reported in Figure 3B. For selected iron-containing food categories (meat and fish products, bread and bakery products, cereal and cereal products, and vegetables and vegetable products) we performed quartile segmentations of high/low consumers of such foods, with the considerations of daily iron intake and serum ferritin levels (Figure 4). Study participants with the lowest intakes (lowest quartile: 0–25%), as well as those with the top intakes (fourth quartile: 75–100%) of meat and fish products, had similar total iron intakes, but notably higher ferritin concentrations were observed in the high consumers of this food category. This was not observed in the other investigated food categories. 

## 4. Discussion

Iron deficiency remains a common global nutritional problem, mainly due to poor iron intake or absorption, and increased body demands in specific population groups, such as women of reproductive age [1], but in Slovenia, this issue has not been previously investigated with a nationally representative study. The results of our study revealed that in all investigated age groups, the mean population-weighted iron intakes were well above the reference values. This was also the case in elderly females, while in adolescent females and adult females up to 50 years, the mean iron intake was slightly below the reference threshold (Table 1). In general, the mean daily iron intakes as reported in the literature are usually higher in males than in females [22], which was also confirmed in our study. However, dietary iron intakes in women of reproductive age in Europe vary considerably among countries, with the lowest mean intakes reported in Bosnia (7.6 mg/day) [73] and the highest in Slovakia (18.9 mg/day) [74]. Studies from European countries show that a considerable proportion (61–97%) of women of reproductive age have a dietary iron intake that is lower than 15 mg/day [37]. In the present study, the proportion of inadequate daily iron intake was 76.3% for adult women of reproductive age and 72.6% in adolescent females. This is notably different compared to males, even when we consider other cut-off values, for example, EFSA’s population reference iron intake values (PRI) or the average requirements (AR) [22] (Table 1). In all age groups and sexes, the proportions of the population not meeting the AR thresholds were very low, meaning that almost the entire population had daily iron intakes of at least the AR values. A histogram of the distribution of daily iron intake (Supplementary Figure S1) in all three investigated population groups showed high variability, which impacted on the mean iron intakes in the population. This could further explain the high proportion of adolescent and adult females with inadequate daily iron intakes, despite the population-weighted mean iron intakes being very close to the recommended daily intake. The males in our study, as was also reported for males in other European countries, had mean dietary iron intakes that were distinctly above the nationally adapted D-A-CH recommendations. For comparison, in Slovakia, the mean iron intakes in males were among the highest in Europe, at 22.7 mg/day [74]. The mean iron intakes of adult males from our study (17.7 mg/day) were comparable to Polish males (17.2 mg/day) [75]. According to the literature data, more than 75% of European males were reported to have iron intakes of above 9 mg/day. In our study, inadequate iron intake was observed in less than 10% of adult and elderly males and in 17.1% of male adolescents (Table 1). It should be noted that regression analysis (Table 2) also highlighted that sex was a significant predictor of dietary iron intake for all age groups. Females had much higher odds for inadequate daily dietary iron intake than males, showing that this problem is much more concerning in the female population, especially in those below 50 years. In the elderly, residential area and financial status were also significantly associated with iron intake; those living outside urban areas and those with higher incomes also had higher iron intakes (Supplementary Table S2). This could be linked to a higher meat consumption.

Dietary iron intakes are closely related to the type of food consumed and reflect dietary habits, as observed in the Slovenian national food consumption study SI.Menu [76,77]. According to the dietary recalls of the above-mentioned study, it has been established that in Slovenia, males tend to consume approximately twice as much meat as compared to females (including processed meat products and offal). As such foods are naturally rich in bioavailable iron, such dietary choices can be reflected in the total daily dietary iron intake, and further in the body iron status. On the other hand, females were recognised as consuming more fruits and vegetables, and were seen as less profuse meat consumers, which could also add to the lower mean daily dietary iron intake in comparison to males. Figure 2 shows that bread and bakery products were the greatest contributors of dietary iron in all population groups. The iron contribution from white bread was quite similar in all age groups, while for brown bread, the elderly had notably greater iron contributions than for other populations. This is because in Slovenia, brown bread is more popular among the elderly [78]. In adults and the elderly, the second largest dietary iron contributor was meat and meat products, but in adolescents, this was cereals and cereal products—particularly breakfast cereals. In Slovenian adolescents, this food category was also found to be an important source of folate [60] and vitamin B12 [72]. This is mainly due to the fact that such foods are often fortified with various vitamins and minerals (e.g., fortified breakfast cereals), including iron [52]. However, cereals are also a natural source of iron, and can have a notable contribution to the daily iron intake, even in non-fortified foods, such as bread and bakery products. Although meat is one of the best sources of bioavailable iron, in Slovenia, bread and related products are typically consumed very frequently, even several times per day, making them a major contributor to dietary iron intake. On the other hand, iron contribution from fruits and vegetables was also notable—particularly from vegetables. Similar findings were also reported in Spain, where the main iron contributors were cereals or grains, meat and derivatives, and vegetables [79]. In an EFSA report on dietary iron contribution from different food categories, a similar pattern can also be observed in other European countries [22].

Iron intake could be also further increased by the consumption of meat and meat products; however, considering the very high meat consumption in Slovenia (particularly in the male population) [76,77], this is not a feasible option. It should be noted that although foods of animal origin are one of the best sources of bioavailable iron, the abundant consumption of non-haem iron-rich foods can result in adequate iron intake and status—also in vegan diets [80]. However, a lower iron bioavailability in such foods can also explain the lower average ferritin status in such populations [81,82]. 

As vegetarians/vegans are not extensively present in the Slovenian population, our study sample size did not allow for the inclusion of this parameter in regression analyses; however, notably higher crude odd ratios for inadequate iron status were observed in vegetarians/vegans in all age groups. Particularly, a high OR for insufficient iron intake was observed in vegetarian/vegan adolescents (OR 14.5; 95%CI 2.1, 624), putting this population at potential risk for iron deficiency (Table 2 and Table S2). The overall evidence shows the importance of a well-balanced diet. Diets with higher intakes of unprocessed cereals, legumes, and other plant foods can have higher iron contributions compared to diets that are high in refined and processed foods, due to their higher iron contents. This is particularly important in individuals with low body iron stores, as in such a case, non-haem dietary iron was reported to be similarly absorbed as haem iron [25,83]. Therefore, meat should not be considered exclusively as the most important contributor of dietary iron, but rather a well-balanced diet, with the inclusion of plenty of unrefined and unprocessed foods being encouraged.

Data on specific biomarkers of body iron status were available only for adults and the elderly, for which blood samples were collected within the Nutrihealth study. Fundamentally, iron balance is regulated by the rate of erythropoiesis and the size of the body iron stores [84]. However, standard biomarkers of iron metabolism include several biomarkers, of which ferritin and serum iron concentration were collected in the present study. Furthermore, haemoglobin levels were measured, indicating the prevalence of anaemia. The population-weighted mean haemoglobin concentration was above the lowest WHO threshold for low haemoglobin (120 g/L) in all population groups (Table 3). Lower population-weighted mean haemoglobin concentrations were seen in females (approximately 140 g/L) in all age groups, and interestingly, they did not differ notably among pre- and post-menopausal females. In males, the mean haemoglobin concentration was approximately 150 g/L. This is comparable to the findings from a study on Slovenian breastfeeding primiparas and males from 2011 [35], where the mean haemoglobin concentrations were 131 g/L and 153 g/L, respectively. With the consideration of menstrual iron losses, the highest prevalence of low haemoglobin concentration could be expected in premenopausal females, but in our study, the highest prevalence was found in females aged 51–65 years, where 6.9% had haemoglobin levels below 120 g/L. 

Various studies report that in premenopausal females, iron deficiency anaemia (IDA) with haemoglobin <120 g/L was present somewhere between 10 and 30% in Europe [50], and the WHO reports that more than 40% of this population has IDA in certain parts of the world, especially in Africa, India, and parts of Asia [85]. In central Europe, for example, a Serbian National Health survey revealed the presence of anaemia according to low haemoglobin concentrations in 27.7% premenopausal females [86]. When considering both low haemoglobin and low ferritin concentrations at once, in females from Italy, Belgium, Germany, and Spain, the anaemia prevalence was found to be 2.9%, 2.2%, 4.1%, and 4.5%, respectively, while in males from the same countries it was mostly below 1% [87].

While our results indicate a better situation in Slovenia, we should highlight the high prevalence of very low ferritin levels in adult premenopausal females (18–50 years); 27.2% had serum ferritin concentrations of below 30 µg/L, and 10.1% were even below 15 µg/L (Table 3), showing depleted body iron stores. According to the WHO criteria [65], such a situation can be already interpreted as a public health concern. In addition, the highest prevalence of serum iron concentration below 13 µmol/L, which is considered as another biomarker for iron deficiency [44], was found in premenopausal females (32%). The population-weighted mean ferritin concentrations in premenopausal females in our study was 68.2 µg/L, which was somewhat higher than that reported in some other studies. The WHO reported ferritin concentration in this population from United Kingdom to range between 30 µg/L and 60 µg/L, and a review of studies from EU countries estimated mean ferritin concentrations at 26–38 µg/L [50]. However, the results of our study show that ferritin concentrations in premenopausal females were often even below 30 µg/L (27.2%), and the median ferritin level was 51.0 µg/L. We should also note that about half of elderly females had ferritin concentrations <100 µg/L, which could, in certain circumstances, mean a greater risk for anaemia in this population, especially as ferritin levels can be increased due to chronic and inflammatory conditions, which are more common among the elderly [88]. Additionally, in males, the risk for anaemia increased with age, starting at approximately 65 years [89]. Ferritin is an established indicator of iron stores, but as it is an acute-phase reactant, its interpretation can be difficult in individuals with accompanying diseases and/or inflammation, where its concentration can be elevated. Nevertheless, in subjects with normal C-reactive protein concentrations, low ferritin is associated with decreased haemoglobin, as has been presented in a Nordic study, which showed that haemoglobin significantly decreased in females and males with ferritin concentrations <20 µg/L and <30 µg/L, respectively [90]. Our study also indicated significantly lower mean haemoglobin concentrations in subjects with ferritin levels of below 30 µg/L (Supplementary Table S6). 

Nevertheless, strong relationships between dietary iron load, its bioavailability, iron requirements, and iron stores [91] need to be mentioned. Dietary factors associated with iron deficiency were also suggested for implementation into blood donor selection processes [92]. On our Nutrihealth study sample we also found significant positive correlation of iron status biomarkers (haemoglobin and ferritin) with meat and fish, and with iron intake from meat and fish, but not with total iron intake (Figure 3B). These results are in line with observations of de Groot [93], who also associated dietary intake of haem iron with both haemoglobin and ferritin levels. We also conducted a regression analyses (Supplementary Table S4), which showed that subjects with adequate iron intakes had significantly lower odds for ferritin levels below 30 µg/L. Similar trends were observed in adult premenopausal females, but the sample of this sub-group was much smaller, which likely affected lower level of significance (*p* > 0.05 and 0.13, respectively). Young et al. also investigated this topic and reported that while both haem and non-haem iron were positively associated with haemoglobin and ferritin levels, haem iron was found to be a much stronger predictor [94]. 

Our results indicate the importance of adequate iron intake; higher iron intake can, at least to some extent, compensate for higher iron requirements and maintain the haemoglobin concentration at a normal level. Due to menstrual losses, premenopausal females are particularly at risk for insufficient iron status [95,96]. Premenopausal females could, in some cases, benefit from iron supplementation to raise haemoglobin and iron stores and reduce the risk for anaemia and symptomatic fatigue caused by low iron status [6,88,97,98]. As iron stores reach a steady state, with iron absorption being adjusted to cover iron losses, even postmenopausal females could possibly benefit from supplementation, depending on the time elapsed from the menopause that is needed to reach consistent iron status [99]. In countries with high rates of iron deficiency, the fortification of certain staple foods is in place, but this also comes with challenges regarding iron bioavailability, and the sensory characteristics of fortified foods [100,101]. Considering our results, such intervention is not needed in Slovenia, where a lower iron status is not common in the overall population but is characteristic particularly in premenopausal females. The regular monitoring of iron status would be beneficial in this population, enabling the implementation of timely individual interventions when necessary.

On the contrary, higher ferritin concentrations were quite common in adult and elderly males, where a very high variability in ferritin concentrations was also observed (Supplementary Figure S2). Mean population-weighted ferritin concentrations >300 µg/L were observed in 17.4% and 22.4% of adult and elderly males, respectively, while less than 5% of females had such ferritin levels. The literature suggests that in males with no inflammation or chronic disease, serum ferritin of greater than 300 µg/L can be indicative of iron overload [66]. The mean population-weighted ferritin concentration was particularly high in elderly males (222.6 µg/L); however, this needs to be interpreted with caution, as inflammation and chronic disease that contribute to a higher ferritin concentration are more common, particularly in the elderly. In addition, a high iron intake, which was observed in Slovenian males, together with higher alcohol consumption, a higher body mass index, and several other factors, can contribute to a high iron status. In case of a long-term iron overload, this could also present a health risk [102,103]. Nevertheless, ferritin level alone could not be used as indicator of iron overload [104]. 

We should also mention concerns that a high intake of haem iron (meat) could increase the risk for cardiovascular disease [105] and cancer [106]. Furthermore, IARC/WHO also highlighted that red meat, and particularly processed meat products, are potentially carcinogenic to humans [107,108], indicating that the consumption of such foods should be limited. Similarly, as in other countries [15,109,110], in Slovenia, we also observed a higher intake of bioavailable haem iron foods in adult males. The dietary iron requirements for males are also lower than for females; therefore, their iron status is expected to be better than in females. We also showed that a higher meat consumption had a greater impact on a higher ferritin status, in comparison to the consumption of other food categories (Figure 4). These observations were also confirmed in correlation analyses between iron status biomarkers and the intake of iron from meats (Figure 3B).

A strength of the present study is that the data on dietary intakes (which included two 24 h dietary recalls and FPQ) were collected for a nationally representative sample of Slovenian adolescents, adults and the elderly, using a standardised EU.Menu methodology [53]. In the scope of the SI.Menu study, we also collected general data on the use of food supplements, but the study was not designed in a way that enabled the estimation of daily iron intake from these sources. Therefore, the study limitation is that iron intake was estimated only from food sources. We should also mention a strength—that we had blood samples available for the subsamples of adults and the elderly (Nutrihealth study), providing insights about markers of body iron status. Unfortunately, blood samples were not available for adolescents. Another limitation of the study design is related to the sampling approach. According to the EU Menu methodology, the SI.Menu study recruited a similar number of adolescents (10–17 years), adults (18–65 years), and the elderly (65–74 years), although the adults subgroup had a much higher age span (47 years) than the other two groups (8 years in adolescents and 11 years in the elderly). Particularly in adults, the creation of additional subgroups (i.e., pre-menopausal women) resulted in relatively small sample sizes. For certain subgroups (e.g., vegetarians/vegans), statistical modelling was also not feasible because of the very low number of these subjects in the population. The study observations for these subgroups should therefore be interpreted with some caution. It should be mentioned that an additional in-depth study is already planned to provide further insights about the population of pre-menopausal women, where vegetarian/vegan subjects will be also targeted as specific population groups. A limitation of the study is also related to the use of biomarkers for body iron status. A key biomarker of iron status used in the present study was ferritin, which is an acute-phase reactant. The limitation is that data on inflammation status (which could be useful in the interpretation of ferritin concentration) were not available.

## 5. Conclusions

In all three investigated population groups, the mean dietary intake of iron in Slovenia was notably higher in males than in females. Among males, the highest usual mean daily iron intake was observed in adults, following by the elderly and adolescents. Among females, all age groups had iron intakes of about 14–15 mg, and insufficient iron intakes were much more prevalent—up to 76% in 18–50-year-old adults females. Sex was also a significant predictor for haemoglobin concentration, both in adults and in the elderly. Low haemoglobin was observed in only a few subjects. On the other hand, depleted iron stores—assessed through the measurement of serum ferritin levels—were particularly prevalent in adult pre-menopausal women, with 27.2% having ferritin concentrations of below 30 µg/L. In comparison to females, males also had higher haemoglobin and serum ferritin concentrations. The main contributors to dietary iron intake were bread and bakery products, meat and meat products, fruits, and vegetables. Particularly, a more extensive consumption of meat and meat products was linked to higher ferritin concentrations. A correlation analyses did not show statistically significant associations between iron status and total dietary iron intakes in any of the investigated population groups, while significant correlations were observed with meat and fish intake, and with iron from meat and fish sources. We can conclude that particularly premenopausal females were identified as the most fragile population in terms of inadequate iron intake and status. Additional research should be considered, particularly in this population group. Such studies should be conducted with larger sample sizes, which should also include sufficient samples of subjects practicing vegan and vegetarian diets. 

## Figures and Tables

**Table 1:** Population-weighted usual daily dietary iron intake and the prevalence of inadequate daily dietary iron intake (95% CI) according to D-A-CH (DRI) and EFSA (PRI, AR) recommendations.

	Adolescents N (%)			Adults N (%)				Elderly N (%)		
	All	Male	Female	All	Male	Female		All	Male	Female
	**10–17 years**	10–17 years	10–17 years	**18–64 years**	18–64 years	18–50 years	51–64 years	**65–75 years**	65–75 years	65–75 years
SI.Menu N (%)	**468 (100)**	238 (50.9)	230 (49.1)	**364 (100)**	173 (47.5)	121 (33.2)	70 (19.3)	**416 (100)**	213 (51.2)	203 (48.8)
**Usual daily iron intake**										
Mean [mg/day] (95%CI)	**16.7** **(16.1–17.2)**	18.4 (17.7–19.2)	14.7 (14.1–15.4)	**16.0** **(15.4–16.5)**	17.7 (16.9–18.6)	14.1 (13.3–15.0)	14.2 (13.1–15.3)	**16.0** **(15.1–16.8)**	17.1 (16.3–17.9)	14.9 (13.6–16.2)
Q25 [mg/day]	**13.1**	14.9	11.6	**12.5**	13.9	11.3	12.1	**12.3**	13.9	11.6
Median [mg/day]	**16.3**	18.0	13.9	**15.5**	17.7	13.8	13.6	**15.5**	17.3	14.0
Q75 [mg/day]	**19.4**	21.0	17.1	**19.2**	20.6	16.6	15.6	**18.7**	18.9	17.5
Mean (95%CI) [mg/1000 kcal/day]	**6.3 (6.1–6.5)**	6.2 (6.0–6.5)	6.4 (6.2–6.6)	**6.3 (6.2–6.5)**	6.4 (6.2–6.6)	6.1 (5.9–6.4)	6.5 (6.2–6.8)	**6.7 (6.5–6.8)**	6.9 (6.7–7.1)	6.5 (6.3–6.7)
**Prevalence of inadequate daily iron intake (%)**										
D-A-CH (DRI) * (95% CI)	**43.7** **(36.7–51.1)**	17.1 (12.1–23.6)	72.6 (64.9–79.1)	**33.4** **(28.3–39.0)**	8.8 (5.2–14.3)	76.3 (67.0–83.6)	22.8 (13.4–36.2)	**13.7** **(10.1–18.4)**	6.1 (3.2–11.3)	20.7 (14.8–28.1)
EFSA (AR) ** (95% CI)	**1.3** **(0.6–2.7)**	0.7 (0.2–2.4)	1.8 (0.6–4.8)	**0.3** **(0.1–1.4)**	0	0.4 (0.01–3.4)	1.2 (0.2–8.0)	**0.1** **(0.01–0.8)**	0.2 (0.03–1.8)	0
EFSA (PRI) *** (95% CI)	**18.6** **(14.6–23.4)**	7.0 (4.1–11.6)	31.1 (23.8–39.5	**29.8** **(24.9–35.2)**	7.3 (4.2–12.4)	69.2 (59.5–77.41)	19.5 (10.7–32.8)	**13.8** **(10.1–18.5)**	7.5 (4.1–13.4)	19.6 (13.9–26.8)

Note: Population weighted for age/sex with consideration of census data. CI: confidence interval; * D-A-CH (DRI) adapted with national recommendations for iron intake (adolescent males: 12 µg/day, adolescent and premenopausal females: 15 µg/day; adult and elderly males, postmenopausal females: 10 µg/day); ** EFSA (AR) average iron requirements (8 µg/day for adolescent males (10–17 years) and females (10–11 years), 7 µg/day for adolescent and premenopausal females, and 6 µg/day for adult males and postmenopausal females). *** EFSA (PRI) population reference iron intake values (11 µg/day for males, postmenopausal and adolescent females (10–11 years); 13 µg/day for adolescent females (12–17 years), and 16 µg/day for premenopausal females (18–50 years)).

**Table 2:** Association between the prevalence of inadequate daily dietary iron intake and different sociodemographic and behavioural variables.

Variable		Adolescents (10–17 Years)			Adults (18–64 Years)			Elderly (65–74 Years)		
		Prevalence (%)	Crude OR (CI)	Adjusted OR (CI)	Prevalence (%)	Crude OR (CI)	Adjusted OR (CI)	Prevalence (%)	Crude OR	Adjusted OR
Unweighted N (%)		208 (44.4)			124 (34.1)			62 (14.9)		
Sex	male	47 (19.8)	1	1	16 (9.3)	1	1	14 (6.6)	1	1
	female *	161 (70.0)	9.48 (6.10–14.87)	10.71 (6.79–16.90)	94 (77.7)	34.16 (16.73–70.96)	51.18 (20.58–127.57)	48 (23.7)	4.40 (2.28–8.94)	3.69 (1.77–7.70)
	female **				14 (20.0)	2.45 (1.03–5.73)	2.67 (1.02–7.02)			
Residential area	rural	112 (41.5)	1	1	61 (30.2)	1	1	28 (12.2)	1	1
	intermediate	35 (46.1)	1.20 (0.70–2.07)	1.15 (0.63–2.11)	21 (37.5)	1.39 (0.71–2.68)	1.50 (0.58–3.88)	12 (16.9)	1.46 (0.63–3.18)	1.04 (0.47–2.32)
	urban	61 (50.0)	1.41 (0.90–2.22)	1.68 (1.00–2.82)	42 (39.6)	1.52 (0.90–2.55)	1.55 (0.73–3.32)	22 (19.0)	1.68 (0.87–3.22)	1.42 (0.70–2.88)
Education	no university degree		n.a.	n.a.	80 (32.1)	1	1	53 (15.5)	1	1
	university degree				44 (38.3)	1.31 (0.80–2.13)	0.65 (0.29–1.46)	9 (12.2)	0.76 (0.31–1.65)	0.52 (0.19–1.38)
Financial status	below average		n.a.	n.a.	35 (29.7)	1	1	44 (16.4)	1	1
	above average				69 (36.5)	1.36 (0.81–2.31)	0.85 (0.39–1.88)	12 (11.2)	0.65 (0.30–1.31)	0.68 (0.32–1.43)
BMI	normal	166 (55.2)	1	1	67 (45.3)	1	1	19 (17.6)	1	1
	overweight	73 (43.7)	0.95 (0.64–1.42)	1.16 (0.74–1.83)	57 (26.4)	0.47 (0.27–0.69)	0.48 (0.24–0.97)	43 (14.0)	0.76 (0.41–1.46)	0.92 (0.46–1.85)
IPAQ	low intensity	38 (35.2)	1	1	39 (30.7)	1	1	15 (11.0)	1	1
	moderate intensity	70 (49.7)	1.82 (1.05–3.14)	1.40 (0.76–2.56)	46 (42.6)	1.67 (0.95–2.97)	1.70 (0.75–3.86)	31 (23.3)	2.47 (1.21–5.20)	1.99 (0.98–4.03)
	high intensity	96 (44.9)	1.50 (0.91–2.49)	1.67 (0.95–2.92)	37 (29.6)	0.95 (0.53–1.68)	0.78 (0.34–1.79)	16 (11.4)	1.05 (0.46–2.39)	0.91 (0.40–2.03)
Employment	employed	n.a.	n.a.	n.a.	80 (35.4)	1	1	n.a.	n.a.	n.a.
	unemployed				18 (42.9)	1.37 (0.65–2.81)	1.96 (0.61–6.34)			
	student				14 (43.8)	1.42 (0.62–3.20)	0.55 (0.14–2.12)			
	retired				12 (18.8)	0.42 (0.19–0.86)	1.45 (1.49–4.31)			
Smoking status	non-smoker	190 (43.4)	1	1	75 (37.7)	1	1	37 (16.0)	1	1
	current, occasional, ex-smoker	18 (60.0)	1.96 (0.87–4.57)	3.66 (1.51–8.88)	49 (29.7)	0.70 (0.44–1.11)	0.74 (0.34–1.52)	25 (13.5)	0.82 (0.45–1.47)	1.18 (0.59–2.35)
Medical diet	no special diet	204 (44.8)	1	1	116 (34.9)	1	1	54 (14.8)	1	1
	medical/weight loss	4 (30.8)	0.55 (0.12–2.00)	0.64 (0.15–2.65)	8 (25.0)	0.62 (0.23–1.49)	0.52 (0.36–1.52)	8 (15.7)	1.07 (0.41–2.47)	1.03 (0.43–2.47)
Behavioural diet	no diet	197 (43.2)	1	n.a.	118 (33.2)	1	n.a.	61 (14.8)	1.	n.a.
	vegetarian/vegan	11 (91.7)	14.46 (2.06–624.7)	n.a.	6 (75.0)	6.05 (1.06–61.87)	n.a.	1 (33.3)	2.88 (0.05–56.02)	n.a.

Notes: n.a.—not applicable; CI: confidence interval; OR: odds ratio; In adolescents and elderly, “female *” included all female participants, while in adults, females were divided into two subgroups: “female *” below 50 years (premenopausal), and “female **” 51–64 years (postmenopausal). Body mass index (BMI) was considered as normal below 25 kg/m^2^, except for adolescents, where gender/age adjusted cut-off points were used. Cut-off odds ratios calculated according to the thresholds of D-A-CH (national) recommendations for iron intake: adolescent males: 12 µg/day, adolescent, and premenopausal females: 15 µg/day; adult and elderly males, postmenopausal females: 10 µg/day. Association was significant for the following variables: *p* < 0.001 sex (adolescents), *p* < 0.01 smoking status (adolescents); *p* < 0.001 sex (adults), *p* < 0.05 BMI (adults), *p* < 0.001 sex (elderly), and *p* = 0.05 IPAQ (elderly).

**Table 3:** Population-weighted mean haemoglobin, serum ferritin, and serum iron concentration, and the prevalence of low haemoglobin, low serum iron, and low/high ferritin for adults and the elderly in the Nutrihealth study (N = 280).

	Adults N (%)				Elderly N (%)		
	All	Male	Female		All	Male	Female
	18–64 years	18–64 years	18–50 years	51–64 years	65–75 years	65–75 years	65–75 years
Nutrihealth N (%)	124 (100)	57 (46.0)	38 (30.6)	29 (23.3)	156 (100)	76 (48.7)	80 (51.3)
Haemoglobin [g/L]							
Mean [g/L] (95%CI)	146.7 (144.3–149.2)	154.0 (151.2–156.9)	139.0 (135.6–142.4)	139.0 (134.4–143.5)	145.1 (143.1–147.1)	152.0 (149.3–154.7)	138.9 (136.7–141.1)
Q25 [g/L]	139.0	149.0	131.0	133.0	136.0	145.0	132.5
Median [g/L]	147.0	153.0	138.0	142.0	145.0	154.0	139.0
Q75 [g/L]	153.0	159.0	144.0	145.0	154.0	159.0	145.5
Prevalence of haemoglobin (%) (95% CI) below 120 and 130 g/L							
<120 g/L	1.1 (0.2–4.3)	0.0	0.0	6.9 (1.7–24.4)	1.3 (0.3–5.2)	1.4 (0.1–9.2)	1.3 (0.1–8.9)
<130 g/L	7.5 (3.9–13.8)	1.3 (0.1–8.7)	14.2 (6.0–30.5)	13.8 (5.1–32.1)	10.3 (6.2–16.4)	4.1 (1.3–12.1)	15.7 (9.1–25.9)
Serum iron [µmol/L]							
Mean (95%CI)	17.9 (16.5–19.2)	18.8 (16.8–20.7)	17.0 (14.7–19.2)	16.1 (14.1–18.1)	19.3 (18.4–20.2)	21.2 (19.8–22.6)	17.6 (16.5–18.8)
Median	17.6	18.4	16.5	14.4	18.8	20.8	16.8
Prevalence of serum iron (%) (95% CI) below 13 µmol/L							
<13 µmol/L	19.9 (13.5–28.4)	10.4 (4.5–22.4)	32.0 (18.8–48.8)	27.6 (14.1–46.8)	8.0 (4.6–13.7)	4.0 (1.3–11.8)	11.7 (6.1–21.1)
Serum Ferritin [µg/L]							
Mean (95%CI)	121.7 (98.7–144.8)	158.1 (121.2–194.9)	68.2 (40.9–95.4)	90.7 (74.3–107.1)	170.5 (144.7–196.2)	222.6 (176.0–269.2)	124.0 (103.2–144.6)
Median	80	121	51	91	126	162	90
Prevalence of serum ferritin (%) (95% CI) below 15, 30, and 100 µg/L, and above 300 and 500 µg/L							
<15 µg/L	3.7 (1.5–8.9)	0	10.1 (3.7–24.6)	3.4 (0.4–21.7)	0.6 (0.1–4.7)	0	1.3 (0.1–8.8)
<30 µg/L	11.6 (7.0–18.5)	3.5 (1.1–10.8)	27.2 (15.3–43.7)	6.9 (1.6–24.4)	7.9 (4.5–13.4)	6.6 (2.7–15.0)	9.1 (4.4–18.0)
<100 µg/L	57.7 (47.8–67.0)	42.3 (28.9–57.0)	86.2 (70.3–94.3)	58.6 (39.9–75.2)	40.2 (32.7–48.1)	26.3 (17.6–37.4)	52.5 (41.5–63.3)
>300 µg/L	10.1 (5.5–17.9)	17.7 (9.3–31.0)	2.4 (0.3–15.9)	0	13.4 (8.9–19.8)	22.4 (14.3–33.2)	5.1 (1.9–13.1)
>500 µg/L	3.4 (1.2–9.7)	5.1 (1.5–16.4)	2.4 (0.3–15.9)	0	3.7 (1.7–8.3)	7.9 (3.6–16.6)	0

Notes: Population weighted for age/sex with consideration of census data. CI: confidence interval.

## Data Availability

The data presented in this study are available on request from the corresponding author.

## Supplementary Materials

The following are available online at https://www.mdpi.com/article/10.3390/nu14235144/s1. Supplementary Table S1.1. Socio-demographic and lifestyle characteristics for dietary SI.Menu study. Supplementary Table S1.2. Demographic characteristics of the Nutrihealth study sample for adult (18–64 years) and elderly (65–74 years) population. Supplementary Table S2. Mean (SD) and model adjusted mean (95% CI) usual daily iron intake by different sociodemographic and behavioural variables for adolescent (10–17 years), adult (18–64 years) and elderly (65–74 years) population. Supplementary Table S3. Relative contribution of selected food categories to usual daily dietary iron intake for adolescent (10–17 years), adult (18–64 years) and elderly (65–74 years) population. Supplementary Table S4. Association between low ferritin concentration (<30 µg/L) and different sociodemographic and behavioural variables in the Nutrihealth study. Supplementary Table S5. Association between ferritin concentration <100 µg/L and different sociodemographic and behavioural variables for elderly population (65–74 years). Supplementary Table S6. Mean (SD) and model adjusted marginal means (95% CI) of haemoglobin according to participants’ sociodemographic, health and dietary related characteristics in adults and the elderly in the Nutrihealth study (N=280). Supplementary Table S7. Mean (SD) total iron intake, iron intake from meat and fish products, ferritin, K-haemoglobin and serum iron for participants in the Nutrihealth study, with exclusion of subjects using iron food supplement. Supplementary Table S8. Contribution of selected food categories to total dietary iron intake according to ferritin status of the participants in the Nutrihealth study. Supplementary Figure S1. Sample population distribution of the reported usual daily dietary iron intake for different age groups (Adolescents: 10–17 years; Adults: 18–64 years; Elderly: 65–74 years). Supplementary Figure S2. Serum ferritin, haemoglobin, and serum iron concentration distribution in adult (18–64 years) and elderly (65–74 years) participants of the Nutrihealth study.
