# Supplementary material for: Dietary Iron Intake and Biomarkers of Iron Status in Slovenian Population: Results of SI.Menu/Nutrihealth Study
Source: Nutrients. 2022 Dec 3;14(23):5144. doi: 10.3390/nu14235144 (PMC9741255; doi:10.3390/nu14235144)
Supplement: Supplementary file 1 [file nutrients-14-05144-s001.zip › nutrients-1980696-supplementary.pdf]

Supplementary Table S1.1. Socio-demographic and lifestyle characteristics for dietary SI.Menu study.

Variable		Adolescents N (%)	Adults N (%)	Elderly N (%)
Overall		468 (100)	364 (100)	416 (100)
Age (mean ± SD)		13.4 (2.37)	43.6 (13.81)	68.7 (2.7)
Residential area	rural	270 (57.7)	202 (55.5)	229 (55.1)
	intermediate	76 (16.2)	56 (15.4)	71 (17.1)
	urban	122 (26.1)	106 (29.1)	116 (27.9)
Sex	male	238 (50.9)	173 (47.5)	213 (51.2)
	female	230 (49.1)	191 (52.5)	203 (48.8)
Education	no university degree	n.a.	249 (68.4)	342 (82.2)
	university degree	n.a.	115 (31.6)	74 (17.8)
Financial status	below average	n.a.	118 (38.4)	269 (71.5)
	above average	n.a.	189 (61.6)	107 (28.5)
Employment status	employed	n.a.	226 (62.1)	n.a.
	unemployed	n.a.	42 (11.5)	n.a.
	student	n.a.	32 (8.8)	n.a.
	retired	n.a.	64 (17.6)	n.a.
BMI (mean ± SD)		21.0 (4.2)	26.7 (5.2)	28.4 (5.0)
BMI	normal	301 (64.6)	148 (40.7)	108 (26.0)
	overweight	167 (35.7)	216 (59.3)	308 (74.0)
Smoking status	current, occasional, ex-smoker	30 (6.4)	165 (45.3)	185 (44.5)
	non-smoker	438 (93.6)	199 (54.7)	231 (55.5)
IPAQ	low intensity	108 (23.3)	127 (35.3)	137 (33.4)
	moderate intensity	141 (30.5)	108 (30.0)	133 (32.4)
	high intensity	214 (46.2)	125 (34.7)	140 (34.2)
Supplement use	user	129 (27.7)	140 (38.4)	95 (22.8)
	non-user	339 (72.3)	224 (61.6)	321 (77.2)
Iron supplement use	user	7 (1.5)	25 (6.9)	5 (1.2)
	non-user	461 (98.5)	339 (93.1)	411 (98.8)
Diet	vegetarian/vegan	12 (2.6)	8 (2.2)	3 (0.7)
	no diet	456 (97.4)	356 (97.8)	413 (99.3)
	medical/weight loss	13 (2.8)	32 (8.8)	51 (12.3)
	no special diet	455 (97.2)	332 (91.2)	465 (87.7)
Chronic disease	not present	/	199 (55.3)	81 (19.7)
	present	/	161 (44.7)	331 (81.3)
Participation in the Nutrihealth study*		/	125 (34.3)	155 (37.3)

Notes: SD: standard deviation; BMI: body mass index; for adults and elderly, normal BMI was considered below 25 kg/m^2, while sex-/age-adjusted cut-off points were used for adolescents; IPAQ—physical activity according to International Physical Activity Questionnaire; *data on biomarkers of iron status were available for participants of the Nutrihealth study. Socio-demographic and lifestyle characteristics for Nutrihealth study is provided in Supplementary Table S1.2. n.a.: Not applicable.

Supplementary Table S1.2. Demographic characteristics of the Nutrihealth study sample for adult (18–64 years) and elderly (65–74 years) population.

Variable		Adults	Elderly
		N (%)	N (%)
		124 (100)	156 (100)
Sex	male	57 (46.0)	76 (48.7)
	female*	38 (30.6)	80 (51.3)
	female**	29 (23.4)	
Residential area	**r**ural	62 (50.0)	84 (53.8)
	**i**ntermediate	20 (16.1)	24 (15.4)
	**u**rban	42 (33.9)	48 (30.8)
Education	no university degree	85 (68.5)	117 (74.5)
	university degree	39(31.5)	39 (25.5)
Financial status	below average	47 (41.6)	93 (62.0)
	above average	66 (58.4)	57 (38.0)
BMI	normal	49 (39.5)	48 (30.1)
	overweight/obese	75 (60.5)	108 (69.9)
IPAQ	low intensity	45 (36.3)	45 (29.0)
	moderate intensity	35 (28.2)	54 (34.8)
	high intensity	44 (35.5)	56 (36.2)
Smoking status	non-smoker	68 (54.8)	92 (59.0)
	current, occasional, ex-smoker	56 (45.2)	64 (41.0)
Chronic disease in the past year	not present	97 (78.2)	101 (64.7)
	present	27 (21.8)	55 (35.3)
Supplement use	user	54 (43.5)	49 (31.4)
	non-user	70 (56.5)	107 (68.6)
iron supplement use	user	7 (5.6)	4 (2.6)
	non-user	117 (94.4)	152 (97.4)
Iron intake	inadequate	36 (30.5)	20 (13.1)
	adequate	82 (69.5)	133 (86.9)
Alcoholic beverage consumption	user	106 (85.5)	134 (85.9)
	non-user	18 (14.5)	22 (14.1)
Medical diet	no special diet	112 (90.3)	145 (87.9)
	medical/weight loss	12 (9.7)	15 (12.1)
Serum ferritin status (< 30 μg/L)	above	107 (86.3)	144 (92.3)
	below	17 (13.7)	12 (7.7)
Serum iron status (< 13 μmol/L)	above	96 (77.4)	142 (91.6)
	below	28 (22.6)	13 (8.4)

Notes: In adolescents and elderly, "female*" included all female participants, while in adults, females were divided into two categories: "female*" below 50 years (premenopausal), and "female**" 51-64 years (postmenopausal). BMI-body mass index; for adults and elderly, normal BMI was considered below 25 kg/m^2, while sex-/age- adjusted cut-off points were used for adolescents; thresholds of adequate/inadequate daily iron intake were based on national D-A-CH recommendations: adolescent males: 12 μg/day, adolescent, and premenopausal females: 15 μg /day, adult and elderly males, postmenopausal females: 10 μg/day; Logistic regression analysis conducted on samples with excluded unreported values (financial status: n = 11 adults, 6 elderly; IPAQ (International Physical Activity Questionnaire): n = 1 elderly, iron intake: n=6 adults, 3 elderly)

Supplementary Table S2. Mean (SD) and model adjusted mean (95% CI) usual daily iron intake by different sociodemographic and behavioural variables for adolescent (10–17 years), adult (18–64 years) and elderly (65–74 years) population.

Variable		Adolescents (10–17 years)		Adults (18–64 years)		Elderly (65–74 years)	
		**Mean (SD)**	**Adjusted mean (CI)**	**Mean (SD)**	**Adjusted mean (CI)**	**Mean (SD)**	**Adjusted mean (CI)**
Sex	male	18.2 (5.7)	18.1 (17.5–18.8)	17.6 (5.1)	17.8 (17.0–18.6)[a]	17.0 (4.8)	16.7 (16.1–17.4)
	female*	14.6 (4.4)	14.8 (14.1–15.4)	13.9 (4.3)	13.5 (12.5–14.5)[b]	14.1 (4.0)	14.4 (13.7–15.1)
	female**			14.3 (4.2)	14.9 (13.6–16.2)[b]		
Residential area	**r**ural	16.5 (5.4)	16.6 (16.0–17.2)	16.0 (5.1)	15.9 (15.2–16.7)	16.2 (4.5)	16.2 (15.5–16.8)[a]
	**i**ntermediate	16.3 (4.8)	16.5 (15.3-17.6)	15.6 (4.8)	15.9 (14.5–17.2)	14.8 (4.4)	15.2 (14.1–16.3)[ab]
	**u**rban	16.3 (5.8)	16.3 (15.4–17.2)	15.3 (5.0)	15.4 (14.4–16.3)	14.9 (4.1)	14.7 (13.8–15.6)[b]
Education	no university degree	n.a.	n.a.	15.5 (4.9)	15.5 (14.9–16.2)	15.5 (4.7)	15.4 (14.9–15.9)
	university degree			16.3 (5.2)	16.1 (15.1–17.2)	16.1 (4.6)	16.3 (15.2–17.5)
Financial status	below average	n.a.	n.a.	15.6 (5.4)	15.8 (14.8-16.7)	15.3 (4.8)	15.2 (14.9–16.8)
	above average			15.9 (4.8)	15.7 (15.0-16.4)	16.4 (4.5)	16.5 (15.6–17.4)
Employment	employed	n.a.	n.a.	16.0 (4.9)	16.0 (15.3–16.7)	n.a.	n.a.
	unemployed			14.3 (4.5)	14.3 (12.6–15.9)		
	student			16.9 (6.0)	17.0 (14.9–19.2)		
	retired			15.1 (4.9)	15.2 (13.7–16.6)		
Smoking status	current, occasional ex-smoker	15.4 (6.3)	15.4 (13.6-17.3)	15.6 (4.8)	15.7 (15.0-16.5)	16.3 (5.1)	15.8 (15.1-16.6)
	non-smoker	16.5 (5.3)	16.6 (16.1-17.0)	15.8 (5.2)	15.7 (14.9-16.6)	15.0 (4.3)	15.4 (14.7-16.0)
BMI	normal	16.7 (5.7)	16.8 (16.3–17.4)	15.9 (5.2)	16.5 (15.6–17.3)	15.0 (4.2)	15.4 (14.4–16.4)
	overweight and obese	16.0 (4.8)	15.8 (15.1–16.6)	15.6 (4.8)	15.3 (14.6–16.0)	15.8 (4.8)	15.6 (15.1–16.2)
IPAQ	low intensity	17.3 (5.7)	17.0 (16.1–18.0)	16.0 (5.1)	16.3 (15.4–17.2)	15.9 (4.7)	15.7 (14.9–16.5)
	moderate	16.0 (5.4)	16.5 (15.7–17.4)	15.3 (4.9)	15.4 (14.4–16.4)	14.9 (4.6)	15.2 (14.4–16.0)
	high intensity	16.4 (5.2)	16.2 (15.5–16.9)	15.7 (4.8)	15.5 (14.6–16.4)	15.9 (4.7)	15.8 (15.0–16.6)

Recent/past disease	not present	n.a.	n.a.	15.9 (5.1)	15.9 (15.2-16.7)	17.1 (8.9)	16.3 (15.2-17.4)
	present			15.5 (4.8)	15.5 (14.7-16.3)	16.1 (4.6)	15.4 (14.9-15.9)
Diet	no special diet	16.4 (5.4)	16.5 (16.0-16.9)	15.7 (4.9)	15.7 (15.2-16.2))	15.6 (4.5)	15.5 (15.0-16.0)
	medical/weight loss	17.5 (5.1)	17.3 (14.5-20.1)	16.2 (6.0)	16.3 (14.5-18.2)	15.6 (5.7)	16.0 (14.7-17.4)
Behavioural diet	no diet	16.6 (5.3)	16.6 (16.2-17.1)	15.7 (5.0)	15.8 (15.2-16.3)	15.6 (4.7)	15.6 (15.2-16.1)
	vegetarian/vegan	9.5 (3.8)	11.4 (8.4-14.3)	15.0 (6.0)	15.2 (11.8-18.6)	13.1 (2.5)	10.8 (5.5-16.2)

Note: In adolescents and elderly, "female*" included all female participants, while in adults, females were divided into two categories: "female*" below 50 years (premenopausal), and "female**" 51-64 years (postmenopausal); BMI-body mass index; for adults and elderly, normal BMI was considered below 25 kg/m2, while sex-/age- adjusted cut-off points were used for adolescents; IPAQ-International Physical Activity Questionnaire; SD-standard deviation; CI-confidence interval; Difference in marginal linear predictions per different socio-demographic and other individual related characteristics within age categories: $p<0.001$ sex (adolescents), $p<0.001$ behavioural diet (adolescents); $p<0.001$ sex (adults), $p<0.05$ BMI (adults); $p<0.001$ sex (elderly), $p<0.05$ residential area (elderly), $p<0.1$ financial status (elderly), $p<0.1$ behavioural diet (elderly).Where three or more levels per predictor are present the pairwise comparisons are performed using Sidak adjustment; different superscript letters indicate significant differences between levels.

Supplementary Table S3. Relative contribution of selected food categories to usual daily dietary iron intake for adolescent (10–17 years), adult (18–64 years) and elderly (65–74 years) population

Food category	Food category contribution in total daily dietary iron intake (%)		
	Adolescents (10-17 years)	Adults (18-64 years)	Elderly (65-74 years)
Fruit and vegetables	15,51	20,57	21,31
Vegetables	10,80	14,23	15,04
Fruit	3,53	4,05	4,59
Nuts and seeds	1,07	2,17	1,51
Bread and bakery products	33,75	34,99	38,89
Bread	26,66	29,35	33,92
White bread	18,49	17,32	16,48
Brown bread	8,17	12,03	17,45
Biscuits	2,85	2,23	1,96
Cakes, muffins, and pastry	4,24	3,41	3,00
Cereal and cereal products	18,82	11,53	7,78
Breakfast cereals	13,92	7,39	4,62
Pasta	3,05	3,13	2,48
Rice	0,68	0,51	0,58
Snackfoods	1,38	0,71	0,29
Convenience foods	2,01	1,88	1,72
Ready meals	2,01	1,88	1,72
Meat and meat products	16,00	19,31	19,36
Processed meat	3,06	3,11	2,78
Unprocessed meat	12,83	15,86	16,16
Fish and fish products	1,59	1,72	1,82
Milk and milk products	3,22	2,62	2,20
Milk	1,95	1,28	1,07
Yogurt	0,35	0,35	0,31
Cheese	0,91	0,97	0,79
Other	7,72	6,67	6,62

Supplementary Table S4. Association between low ferritin concentration (<30 μg/L) and different sociodemographic and behavioural variables in the Nutrihealth study

Variable	Level	Adults (18–64 years old)			Elderly (65–74 years old)		
		Prevalence (%)	Crude OR (CI)	Adjusted OR (CI)	*Prevalence (%)*	Crude OR (CI)	Adjusted OR (CI)
Unweighted data N (%)		17 (13.7)			12 (7.7)		
Sex	male	4 (7.0)	1	1	5 (6.6)	1	1
	female	13 (9.4)	3.2 (0.9-14.2)	3.5 (0.3-47.2)	7 (8.8)	1.4 (0.4-5.7)	1.6 (0.4-7.2)
Residential area	**r**ural	6 (9.7)	1	1	5 (6.0)	1	1
	**i**ntermediate	3 (15.0)	1.6 (0.2-8.7)	1.4 (0.1-14.6)	1 (4.2)	0.7 (0.01-6.6)	0.3 (0.2-4.6)
	**u**rban	8 (19.1)	2.2 (0.6-8.3)	1.2 (0.2-6.2)	6 (12.5)	2.3 (0.5-9.9)	1.9 (0.4-8.4)
Education	no university degree	9 (11.4)	1	1	6 (5.3)	1	1
	university degree	7 (18.0)	1.7 (0.5-5.6)	1.8 (0.3-10.9)	6 (15.4)	3.3 (0.8-13.0)	3.8 (0.8-17.1)
Financial status	below average	6 (13.6)	1	1	7 (7.6)	1	1
	above average	8 (12.7)	0.9 (0.3-3.5)	0.4 (0.1-2.6)	5 (9.1)	1.2 (0.3-4.7)	1.3 (0.3-5.6)
BMI	normal	12 (26.1)	1	1	2 (4.4)	1	1
	overweight	4 (5.6)	0.2 (0.04-0.6)	0.2 (0.04-1.1)	10 (9.4)	2.3 (0.5-22.0)	3.0 (0.5-17.3)
IPAQ	low intensity	3 (7.1)	1	1	5 (11.1)	1	1
	moderate intensity	6 (17.7)	2.8 (0.5-18.4)	2.4 (0.3-19.1)	3 (5.6)	0.5 (0.1-2.6)	0.3 (0.1-1.7)
	high intensity	7 (16.7)	2.6 (0.5-16.6)	6.0 (0.8-45.9)	4 (7.6)	0.7 (0.1-3.3)	0.6 (0.1-2.9)
Smoking status	non-smoker	8 (12.7)	1	1	8 (9.0)	1	1
	current, occasional, ex-smoker	8 (14.6)	1.2 (0.4-3.9)	0.7 (0.1-3.6)	4 (6.3)	0.7 (0.1-2.7)	0.5 (0.1-2.4)
Chronic disease in the past year	not present	14 (14.4)	1	1	8 (7.9)	1	1
	present	3 (11.1)	0.7 (0.1-3.0)	1.3 (0.2-9.8)	4 (7.3)	0.9 (0.2-3.6)	1.0 (0.2-4.8)
Supplement use	non-user	6 (9.2)	1	1	9 (8.7)	1	1
	user	10 (18.9)	2.3 (0.7-8.2)	2.1 (0.4-9.7)	3 (6.1)	0.7 (0.1-2.9)	0.4 (0.1-1.8)
Iron intake	inadequate	10 (27.8)	1	1	2 (10.0)	1	1
	adequate	6 (7.3)	0.2 (0.1-0.7)	0.2 (0.3-1.0)	10 (7.5)	0.7 (0.1-7.4)	0.4 (0.1-2.9)
Tea consumption	<3x week/never	15 (14.2)	1	1	10 (7.1)	1	1
	other	2 (11.1)	0.8 (0.1-3.8)	0.3 (0.2-3.6)	2 (13.3)	0.5 (0.01-4.0)	2.1 (0.3-13.3)
Alcoholic beverage	users	14 (13.2)	1	1	11 (8.2)	1	1

consumption	non-users	3 (16.7)	1.3 (0.2-5.6)	0.4 (0.3-22.5)	1 (4.6)	2.0 (0.2-11.03)	0.5 (0.04-4.8)
Medical diet	no special diet	15 (14.1)	1	1	10 (7.3)	1	1
	medical/weight loss	1 (9.1)	0.6 (0.01-4.9)	0.8 (0.3-18.1)	2 (13.3)	1.07 (0.41-2.47)	2.9 (0.4-24.2)
Behavioural diet	no diet	15 (12.9)	1	n.a.	12 (8.0)	n.a.	n.a.
	vegetarian/vegan	1 (50)	6.7 (0.1-533.5)	n.a.	0 (0.0)	n.a.	n.a.

Notes: Cut-off odds ratios (OR) calculated at <30 μg/L serum ferritin concentration. n.a. not applicable; CI: confidence interval; OR: odds ratio; BMI: body mass index; normal considered below 25 kg/m²; thresholds of adequate/inadequate daily iron intake were based on nationally adapted D-A-CH recommendations: adolescent males: 12 μg/day, adolescent, and premenopausal females: 15 μg /day, adult and elderly males, postmenopausal females: 10 μg/day; IPAQ-International Physical Activity Questionnaire; Significant association: p<0.05 iron intake (adults); Marginally significant association: p<0.1 education (elderly).

Supplementary Table S5. Association between ferritin concentration <100 μg/L and different sociodemographic and behavioural variables for elderly population (65–74 years)

Variable	Level	Elderly (65–74 years)		
		Prevalence (%)	Crude OR (CI)	Adjusted OR (CI)
Unweighted data N (%)		65 (41.7)		
Sex	male	21 (27.6)	1	1
	female	44 (55.0)	3.2 (1.6-6.6)	2.9 (1.3-6.6)
Residential area	**r**ural	31 (36.9)	1	1
	**i**ntermediate	11 (45.8)	1.4 (0.5-4.0)	1.1 (0.4-3.2)
	**u**rban	23 (47.9)	1.6 (0.7-3.4)	1.4 (0.6-3.3)
Education	no university degree	42 (36.8)	1	1
	university degree	20 (51.3)	1.8 (0.8-4.0)	1.4 (0.6-3.5)
Financial status	below average	33 (35.9)	1	1
	above average	26 (47.3)	1.6 (0.8-3.3)	1.0 (0.4-2.3)
BMI	normal	19 (17.6)	1	1
	overweight/obese	43 (14.0)	0.6 (0.3-1.2)	0.8 (0.4-1.8)
IPAQ	low intensity	21 (46.7)	1	1
	moderate intensity	22 (40.7)	0.8 (0.3-1.9)	0.9 (0.4-2.1)
	high intensity	18 (34.0)	0.6 (0.2-1.4)	0.6 (0.3-1.6)
Smoking status	non-smoker	39 (43.8)	1	1
	current, occasional, ex-smoker	23 (35.9)	0.7 (0.4-1.5)	1.2 (0.5-2.7)
Recent/past disease	not present	44 (43.6)	1	1
	present	21 (38.2)	0.8 (0.4-1.6)	0.8 (0.3-1.7)
Supplement use	non-user	39 (37.5)	1	1
	user	23 (46.9)	1.5 (0.7-3.1)	1.0 (0.4-2.2)
Iron intake	inadequate	9 (45.0)	1	1
	adequate	53 (39.9)	0.8 (0.3-2.4)	1.1 (0.4-3.2)
Tea consumption	<3x week/never	58 (41.1)	1	1
	other	7 (46.7)	1.3 (0.4-4.2)	1.8 (0.6-5.9)
Alcoholic beverage consumption	users	55 (41.0)	1	1
	non-users	10 (45.5)	1.2 (0.4-3.3)	1.2 (0.5-3.9)
Medical diet	no special diet	54 (39.1)	1	1
	medical/weight loss	8 (53.3)	1.8 (0.5-6.1)	2.6 (0.8-9.1)

Notes: Cut-off odds ratios (OR) calculated at <100 μg/L serum ferritin concentration. CI: confidence interval; BMI: body mass index; considered as normal below 25 kg/m^2, thresholds of adequate/inadequate daily iron intake were based on nationally adapted D-A-CH recommendations for elderly: 10 μg/day; IPAQ-International Physical Activity Questionnaire; Significant correlation: $p < 0.05$ sex.

Supplementary Table S6. Mean (SD) and model adjusted marginal means (95% CI) of haemoglobin according to participants' sociodemographic, health and dietary related characteristics in adults and the elderly in the Nutrihealth study (N=280).

Variable		Adults (18–64 years)		Elderly (65–74 years)	
		N (%)	Adjusted mean (CI)	N (%)	Adjusted mean (CI)
		124 (100)		156 (100)	
Sex	male	57 (46.0)	152.5 (148.8-156.2)	76 (48.7)	150.2 (147.6-152.8)
	female*	38 (30.6)	140.8 (136.2-145.3)	80 (51.3)	140.8 (138.2-143.3)
	female**	29 (23.4)	139.3 (135.2-143.4)		
Residential area	rural	62 (50.0)	144.9 (142.0-147.8)	84 (53.8)	146.4 (144.1-148.7)
	intermediate	20 (16.1)	145.5 (140.6-150.4)	24 (15.4)	140.7 (136.2-145.1)
	urban	42 (33.9)	144.8 (141.5-148.2)	48 (30.8)	145.9 (142.7-149.1)
Education	no university degree	85 (68.5)	145.4 (142.9-148.0)	117 (74.5)	145.4 (143.4-147.4)
	university degree	39(31.5)	143.9 (140.2-147.7)	39 (25.5)	145.4 (141.7-149.1)
Financial status	below average	47 (41.6)	142.1 (138.7-145.4)	93 (62.0)	144.8 (142.6-147.0)
	above average	66 (58.4)	146.9 (144.2-149.6)	57 (38.0)	146.4 (143.4-149.3)
BMI	normal	49 (39.5)	144.4 (141.0-147.7)	48 (30.1)	144.2 (141.0-147.4)
	overweight	75 (60.5)	145.3 (142.7-147.9)	108 (69.9)	145.9 (143.9-147.9)
IPAQ	low intensity	45 (36.3)	143.5 (140.1-146.9)	45 (29.0)	146.6 (143.4-149.7)
	moderate intensity	35 (28.2)	145.8 (142.2-149.3)	54 (34.8)	144.7 (141.8-147.5)
	high intensity	44 (35.5)	145.7 (142.2-149.1)	56 (36.2)	145.2 (142.3-148.1)
Smoking status	non-smoker	68 (54.8)	143.5 (140.8-146.3)	92 (59.0)	144.4 (142.8-147.0)
	current, occasional, ex-smoker	56 (45.2)	146.5 (143.6-149.4)	64 (41.0)	146.9 (144.0-149.7)
Recent/past disease	not present	97 (78.2)	144.6 (142.3-146.8)	101 (64.7)	144.9 (142.8-147.0)
	present	27 (21.8)	146.1 (141.9-150.3)	55 (35.3)	146.3 (143.3-149.3)
Supplement use	user	54 (43.5)	144.0 (140.8-147.2)	49 (31.4)	145.3 (142.2-148.4)
	non-user	70 (56.5)	145.7 (142.9-148.4)	107 (68.6)	145.4 (143.3-147.5)
Iron intake	inadequate	36 (30.5)	146.5 (141.9-151.1)	20 (13.1)	143.6 (139.0-148.3)
	adequate	82 (69.5)	144.0 (141.4-147.0)	133 (86.9)	145.7 (143.9-147.5)
Alcoholic beverage consumption	users	106 (85.5)	144.0 (141.9-146.1)	134 (85.9)	144.8 (143.0-146.6)
	non-users	18 (14.5)	149.7 (144.5-154.9)	22 (14.1)	149.0 (144.3-153.7)
Medical diet	no special diet	112 (90.3)	144.8 (142.8-146.8)	145 (87.9)	146.5 (144.7-148.2)
	medical/weight loss	12 (9.7)	146.0 (139.8-152.2)	15 (12.1)	135.6 (129.9-141.3)
Serum ferritin	≥ 30 μg/L	107 (86.3)	146.6 (144.5-148.7)	144 (92.3)	146.2 (144.5-147.9)
	< 30 μg/L	17 (13.7)	134.1 (128.1-140.1)	12 (7.7)	136.9 (130.9-142.9)
Serum iron	≥ 13 μmol/L	96 (77.4)	146.2 (144.0-148.5)	142 (91.6)	146.1 (144.3-147.8)
	< 13 μmol/L	28 (22.6)	140.8 (136.5-145.1)	13 (8.4)	138.1 (132.1-144.0)

Notes: In adolescents and elderly, "female*" included all female participants, while in adults, females were divided into subgroup of "female*" below 50 years (premenopausal), and "female**" 51-64 years (postmenopausal). SD: standard deviation; CI: confidence interval; BMI: body mass index (considered as normal below 25 kg/m2); thresholds of adequate/inadequate iron intake were based on nationally adapted D-A-CH recommendations: adolescent males: 12 μg/day, adolescent and premenopausal females: 15 μg /day; adult and elderly males, postmenopausal females: 10 μg/day. Association based on contrast of marginal linear predictions was significant for the following variables: p<0.001 sex (adults), p<0.05 education (adults), p=0.05 alcoholic beverage consumption (adults), p<0.001 ferritin status (adults), p<0.05 serum iron status (adults); p< 0.001 sex (elderly), p<0.001 medical diet (elderly), p<0.001 ferritin status (elderly), and p<0.05 serum iron status (elderly). Marginal correlation with p<0.1 was found for residential area (elderly) and alcoholic beverage consumption (elderly).

Supplementary Table S7. Mean (SD) total iron intake, iron intake from meat and fish products, ferritin, K-haemoglobin and serum iron for participants in the Nutrihealth study, with exclusion of subjects using iron food supplement.

Variables	Levels	Total iron intake (mg/day)	Iron intake from meat and fish products (mg/day)	Ferritin [μg/L]	Haemoglobin [g/L]	Serum iron [μmol/L]
		Mean (SD)	Mean (SD)	Mean (SD)	Mean (SD)	Mean (SD)
Sex/Age	Male (18-64 y.)	17.7 (4.3)	3.5 (1.4)	179 (151.2)	152.1 (10.4)	18.8 (6.3)
	Male (64-75 y.)	16.7 (4.6)	3.0 (1.4)	222.6 (206.2)	152 (11.6)	20.9 (6.6)
	Female (18-50 y.)	14.5 (4.3)	2.0 (0.9)	70.3 (92.9)	139.3 (10.1)	16.6 (6.8)
	Female (50-64 y.)	15.0 (4.9)	2.8 (2.5)	91.2 (41.7)	140.4 (9.7)	16.6 (4.8)
	Female (64-75 y.)	15.1 (4.2)	2.1 (1.2)	125.2 (96.7)	138.7 (9.7)	17.7 (5.1)
BMI	normal	16 (4.7)	2.4 (1.3)	125.1 (126.5)	143.6 (12)	18.8 (6.8)
	overweight/obese	15.9 (4.5)	2.8 (1.6)	167.5 (163.3)	146.3 (12.2)	18.5 (5.9)
Residential area	rural	16.1 (4.4)	2.8 (1.4)	162.5 (166.8)	146.6 (13.1)	18.4 (6)
	intermediate	15.5 (4.1)	2.7 (1.3)	161.1 (132.1)	143.1 (11.5)	17.4 (5.2)
	urban	15.9 (4.9)	2.5 (1.8)	134.7 (137.2)	144.3 (10.7)	19.5 (6.7)
Education	no university degree	16.1 (4.8)	2.7 (1.6)	154 (156.9)	145.3 (12.2)	18.9 (6.1)
	university degree	15.7 (3.8)	2.8 (1.4)	150.9 (143.4)	145.5 (12.2)	17.8 (6.2)
Financial status	below average	15.8 (4.8)	2.7 (1.7)	157.4 (155.9)	144.7 (11.6)	18.3 (5.3)
	above average	16 (4.4)	2.6 (1.3)	145.7 (133.9)	146.1 (12.7)	19 (7.2)
IPAQ	low intensity	16.1 (4.8)	2.8 (1.9)	167.1 (187.0)	145.6 (13.1)	17.5 (5.3)
	moderate	15.9 (4.8)	2.6 (1.1)	145.2 (137.5)	145.0 (11.2)	18.6 (6.6)
	high intensity	16.0 (4.1)	2.7 (1.5)	153.9 (138.2)	146.4 (12.4)	19.8 (6.5)
Smoking status	non-smoker	15.9 (4.5)	2.6 (1.6)	144.2 (145.1)	142.7 (11.6)	17.5 (5.3)
	current, occasional, ex-smoker	16.1 (4.7)	2.7 (1.4)	164.7 (162.3)	148.8 (12.2)	20 (6.9)
Chronic disease in the past year	not present	16 (4.6)	2.7 (1.6)	134.3 (132.2)	145 (12)	18.3 (6.5)
	present	15.9 (4.4)	2.6 (1.2)	196.1 (185.9)	146.2 (12.8)	19.2 (5.4)

Notes: Total iron intake with foods (not including food supplements and medicines); SD: standard deviation; BMI: body mass index; considered as normal below 25 kg/m^2.

Supplementary Table S8. Contribution of selected food categories to total dietary iron intake according to ferritin status of the participants in the Nutrihealth study.

Food category	Contribution of the food category to dietary iron intake			
	Ferritin concentration <30 µg/L Mean (SD)	Ferritin concentration >30 µg/L Mean (SD)	Ferritin concentration <100 µg/L Mean (SD)	Ferritin concentration >100 µg/L Mean (SD)
Vegetable and vegetable products (%)	10.7 (5.6)	10.8 (6.7)	10.5 (6.3)	11.2 (6.9)
Cereal and cereal products (%)	11.5 (8.4)	9.4 (9.7)	10.6 (10.8)	8.7 (8.1)
Bread and bakery products (%)	27.2 (11.5) [a]	30.3 (11.7) [b]	28.7 (11.7)[a]	31.2 (11.6)[b]
Meat and fish products (%)	17.8 (7.3)	18.2 (8.2)	17.3 (7.7)[a]	19.0 (8.4)[b]
Mean dietary iron intake (µg/day)	15.7 (4.7)	15.9 (4.5)	15.6 (4.3)	16.2 (4.7)

Note: Superscript letters denote marginally significant difference at $p < 0.1$ using t-test for independent samples.

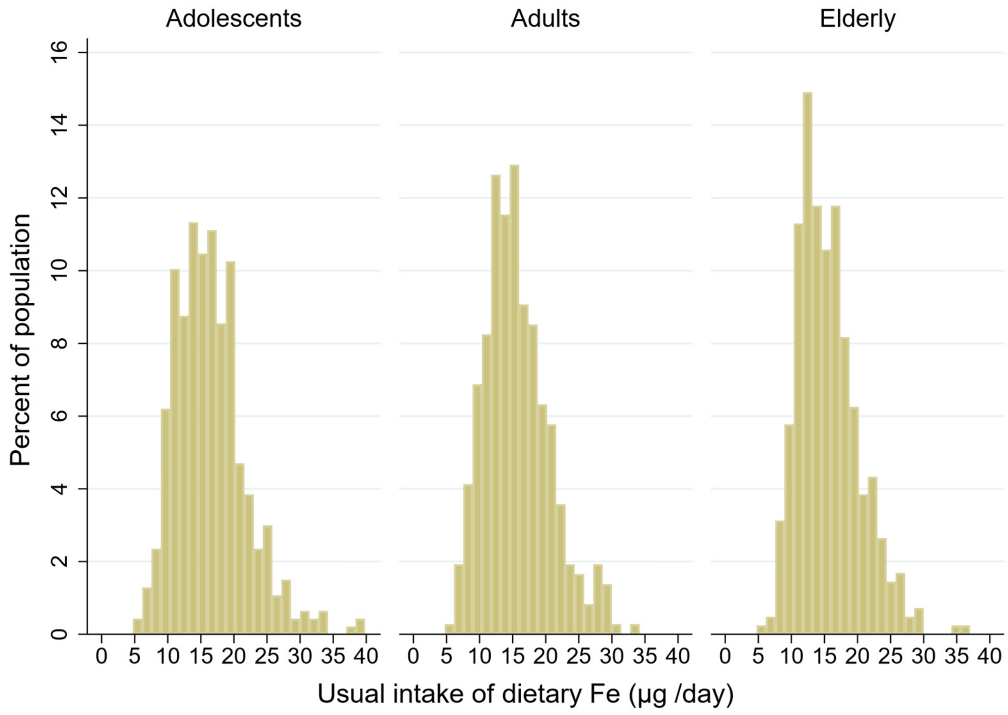

Supplementary Figure S1. Sample population distribution of the reported usual daily dietary iron intake for different age groups (Adolescents: 10-17 years; Adults: 18-64 years; Elderly: 65-74 years).

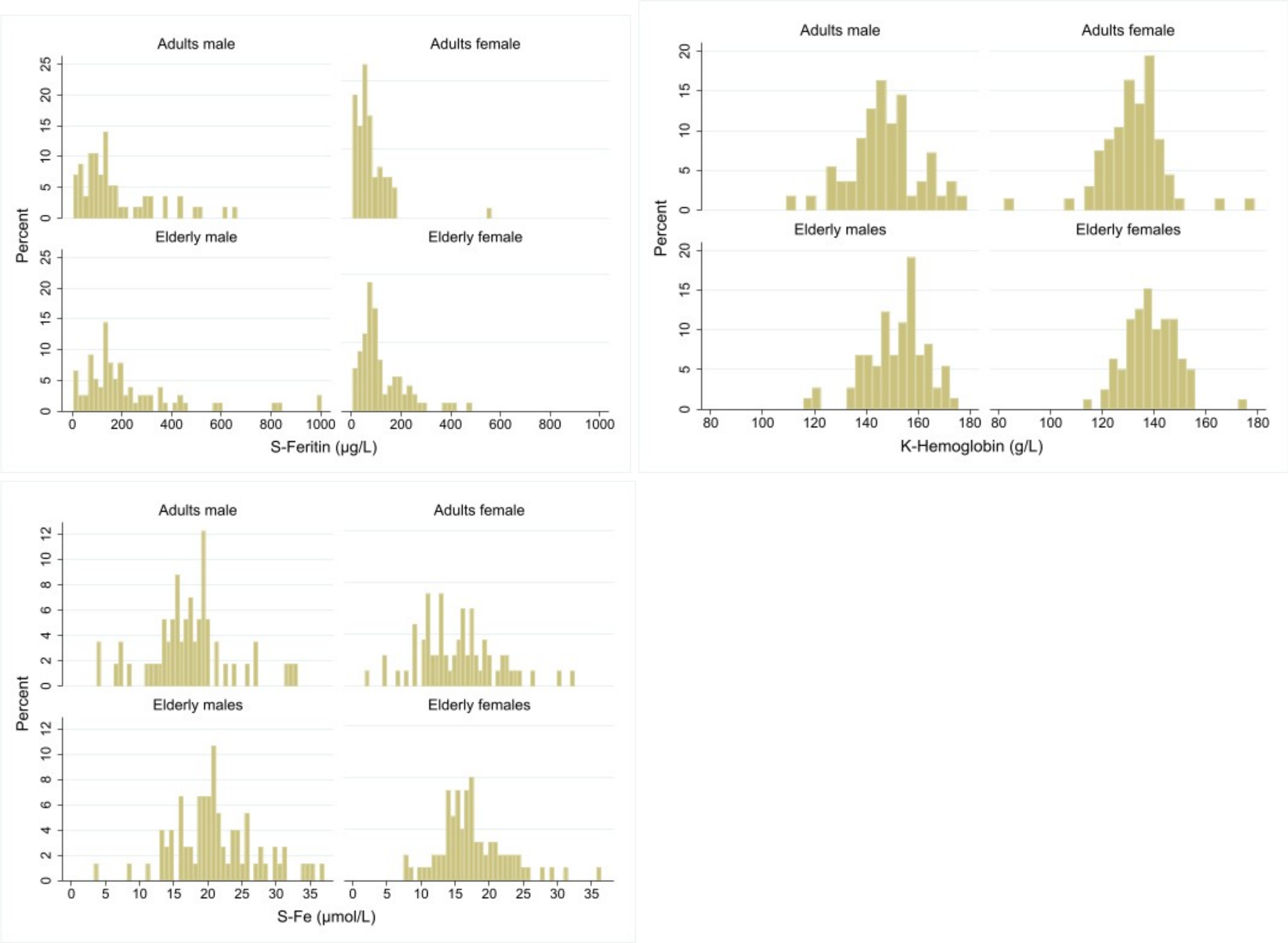

Supplementary Figure S2. Serum ferritin, haemoglobin, and serum iron concentration distribution in adult (18-64 years) and elderly (65-74 years) participants of the Nutrihealth study.
